# Structural basis for modulation of group III mGlu receptors by transsynaptic interactions

**DOI:** 10.1126/sciadv.aed3793

**Published:** 2026-09-04

**Authors:** William G. Ludlam, Chu-Ting Chang, Kristina Cechova, Brandon W. Liauw, Hwa-Jin Cho, Safoura Salar, Anjelique Sawh-Gopal, Afroza Parvin, Simrat K. Dhaliwal, Simran K. Dhaliwal, Tina Izard, Huan Bao, Anne M. Brown, Henry A. Dunn, Reza Vafabakhsh, Kirill A. Martemyanov

**Affiliations:** ^1^ Department of Physiology and Biophysics, University of Miami, Miller School of Medicine, Miami, FL 33136, USA.; ^2^ Skaggs Graduate School, The Scripps Research Institute, Jupiter, FL 33458, USA.; ^3^ Department of Molecular Biosciences, Northwestern University, Evanston, IL 60208, USA.; ^4^ The Herbert Wertheim UF Scripps Institute for Biomedical Innovation and Technology, University of Florida, Jupiter, FL 33458, USA.; ^5^ Department of Pharmacology and Therapeutics, University of Manitoba; and Division of Neurodegenerative and Neurodevelopmental Disorders, St. Boniface Hospital Albrechtsen Research Centre, Winnipeg, Manitoba, Canada.; ^6^ Department of Molecular Physiology and Biological Physics, University of Virginia, Charlottesville, VA 22903, USA.; ^7^ Department of Biochemistry, Virginia Tech, Blacksburg, VA 24061, USA.

## Abstract

Group III metabotropic glutamate receptors (mGluRs) are critical signaling molecules that regulate strength, homeostasis, and plasticity of glutamatergic synaptic signaling. These receptors are engaged in transsynaptic interactions with extracellular leucine-rich repeat and fibronectin type III domain-containing (ELFN) cell adhesion proteins. ELFN proteins have been shown to play a critical role in regulation of activity and localization of mGluRs activity in vivo, yet the exact nature of their regulatory interaction has remained unknown. Here, we present a cryo–electron microscopy structure of the ELFN-mGluR complex. We identify a specific ELFN-binding pocket on mGluRs involved in its allosteric regulation through the network of residues affecting the orthosteric ligand binding site. We further uncover cooperativity whereby mGluR activation increases their association with ELFN proteins as a potential feedback mechanism to regulate synaptic strength. Last, we determine that disruption in mGluR-ELFN interaction is a recurring mechanism underlying several neurological conditions as we delineate their structure-functional etiology.

## INTRODUCTION

Glutamate is the primary excitatory neurotransmitter of the brain. Release of glutamate at neuronal synapses is a crucial mechanism underlying many brain functions, including learning and motor control. Thus, for proper brain function, glutamatergic synapses maintain tight spatial and temporal control over glutamate release. Group III metabotropic glutamate receptors (mGluRs) that include mGluR4, mGluR6, mGluR7, and mGluR8 constitute a family of G protein–coupled receptors (GPCRs) that mostly inhibit presynaptic release of glutamate. In the central nervous system, mGluR4, mGluR7, and mGluR8 serve as autoreceptors that limit glutamate release upon excessive stimulation and provide a basis for short-term synaptic plasticity (*1*). In the retina, mGlu6 serves as the principal receptor enabling synaptic communication of photoreceptors (*2*). Recently, it has been discovered that presynaptic group III mGluRs are targeted to synapses through transsynaptic interactions with cell adhesion molecules: extracellular leucine-rich repeat and fibronectin type III domain-containing (ELFN) family members ELFN1 and ELFN2 (*3*–*8*). However, the structural organization and functional regulation of complex assembly remain unknown.

A growing body of evidence suggests that perturbations in group III mGluR signaling are implicated in a wide spectrum on neurodevelopmental disorders (NDDs) with links to autism, intellectual disabilities, movement disorders, and schizophrenia (*9*–*13*). In particular, missense mutations in mGluR7 cause GRM7-related disorder, characterized by varying neurological symptoms that include seizures, developmental delays and motor abnormalities among others (*14*). Loss of ELFN produces a similar profile of nervous system dysfunction in both mice and humans. In humans, genetic variations in ELFN1 have been causally linked to a NDD that features seizures, hyperactivity, developmental delays, and autism (*3*, *15*–*18*). Knockout of ELFN1 or ELFN2 in mice results in neuropsychiatric manifestations such as seizure susceptibility, hyperactivity, anxiety-like symptoms, and deficits in social interaction (*3*, *5*, *15*, *17*). Additionally, ELFN1 and ELFN2 play critical roles in wiring rod and cone photoreceptors, respectively, and their knockout in mice abolishes synaptic transmission of signal generated by photoreceptors in response to light, resulting in night blindness (*19*, *20*).

Mechanistically, ELFN proteins regulate mGluR activity by stabilizing mGluR at synapses (*3*, *5*, *7*, *20*) and enhancing constitutive mGluR basal activity (*5*, *6*), thereby increasing presynaptic glutamate release probability (*7*, *21*). Intriguingly, ELFNs also exert an allosteric modulatory effect on group III mGluR activity (*4*, *5*, *8*, *20*). However, the mechanisms underlying the effect of ELFN proteins on mGluRs are not well understood.

In this work, we report the structure of an mGluR-ELFN complex obtained by cryo–electron microscopy (cryo-EM) and apply the insights generated to probe the molecular basis of group III mGluRs modulation by ELFN proteins. We then use this information to solve the mechanisms by which mutations in mGluR and ELFN proteins lead to nervous system disorders.

## RESULTS

### Architecture of the mGluR7-ELFN2 complex

To understand the structural basis behind regulation of group III mGluRs by ELFN proteins, we focused on the mGluR7-ELFN pair given its potential for cross-class generalization. While all group III mGluRs can interact with both ELFN1 and ELFN2 (*4*, *5*, *20*), the mGluR7 subtype is the most highly expressed in the brain and is also the most evolutionarily conserved (*1*, *22*). mGluR7 is also an attractive model for structural studies because it is the only subtype for which an agonist-free structure has been solved (*23*). Similarly, of the two ELFN proteins, ELFN2 is more broadly expressed and has been shown to control all members of the group III mGluR family in vivo (*5*, *24*).

**Fig. 1.**
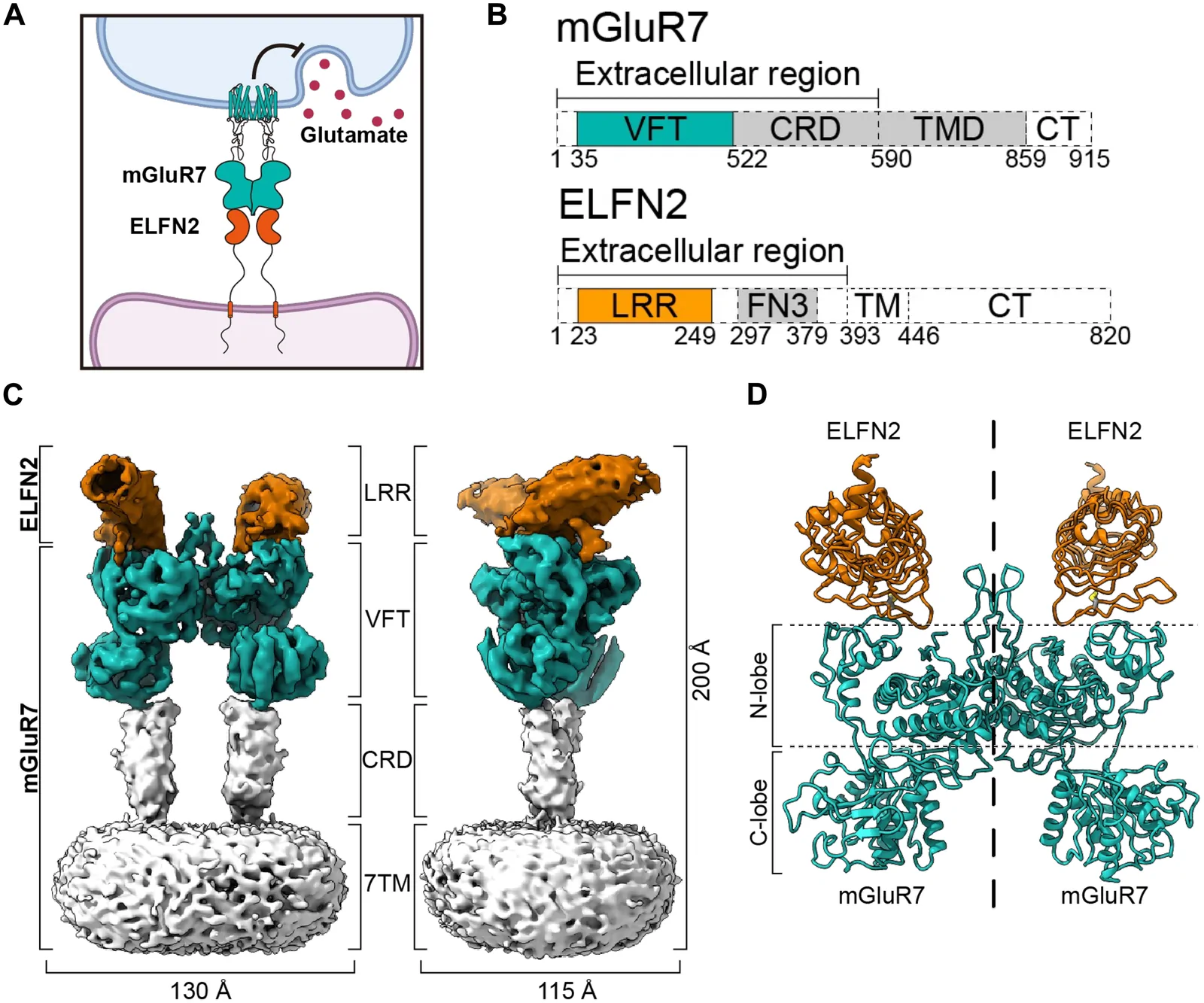
Architecture of the mGluR7-ELFN2 complex. (**A**) Postsynaptic ELFN2 forms a transsynaptic connection by interacting with presynaptic mGluR7. Activation of mGluR7 inhibits release of glutamate vesicles into the synapse. (**B**) Domain architecture of mGluR7 includes an N-terminal signal peptide, Venus flytrap (VFT), cysteine-rich domain (CRD), seven transmembrane (TMD), and intracellular C terminus (CT). ELFN2 domain architecture consists of an N-terminal signal peptide, leucine-rich repeat (LRR) domain, fibronectin 3 (FN3) domain, a single-pass transmembrane domain (TM), and an intracellular C terminus (CT). (**C**) Cryo-EM map of mGluR7-ELFN2 complex in lipid micelle. ELFN2 LRR domain in dark orange and mGluR7 VFT domain in dark blue. (**D**) Ribbon model of mGluR7 VFT in complex with ELFN2 LRR.

For the structural studies, we reconstituted purified recombinant mGluR7 protein in native nanodiscs using peptide scaffolds (*25*) with the soluble ectodomain of ELFN2 in the presence of the compound MMPIP, as used in the previous structural determination of inactive mGluR7 (*23*) and applied cryo-EM to build a three-dimensional (3D) model of the complex (fig. S1, A to C). After classification, we identified a major species present in the sample that resembled the known structure of the inactive mGluR7 dimer [Protein Data Bank (PDB): 7EPC] with two additional densities present that resembled the leucine-rich repeat (LRR) domains of ELFN2 (Fig. 1, A to C). These densities were connected apically to each of the N-terminal lobes of the ligand-binding Venus-flytrap (VFT) domain. Although the fibronectin 3 domain was also present in our ecto-ELFN2 construct, we did not observe any density for it in our particles. We observed density for the cysteine-rich domain (CRD; or stalk region) on mGluR7 but could not achieve enough resolution to unambiguously dock the protein backbone. Within the lipid particle, we could not distinguish any density to determine the position of the seven transmembrane (7TM) domains, possibly due to anisotropic angular distribution of particles (fig. S1D). We were able to refine the structural model to 4.6-Å resolution in the VFT domain (fig. S1E), allowing us to define the sequence of the mGluR7 VFT domain and ELFN2 LRR domain within the density. The highest resolution was achieved in the upper lobe of the mGluR7 VFT domain adjacent to where ELFN2 binds (fig. S1F). In these regions of highest resolution, we could dock the side chains of bulky residues. The resulting model revealed a dimer-of-dimer configuration with C2 symmetry (Fig. 1D). Notably, each ELFN2 molecule contacted each of the mGluR7 protomers without making intermolecular contacts between the LRR domains resolved in the structure.

### Structural basis for selective interaction of group III mGluR with ELFN proteins

**Fig. 2.**
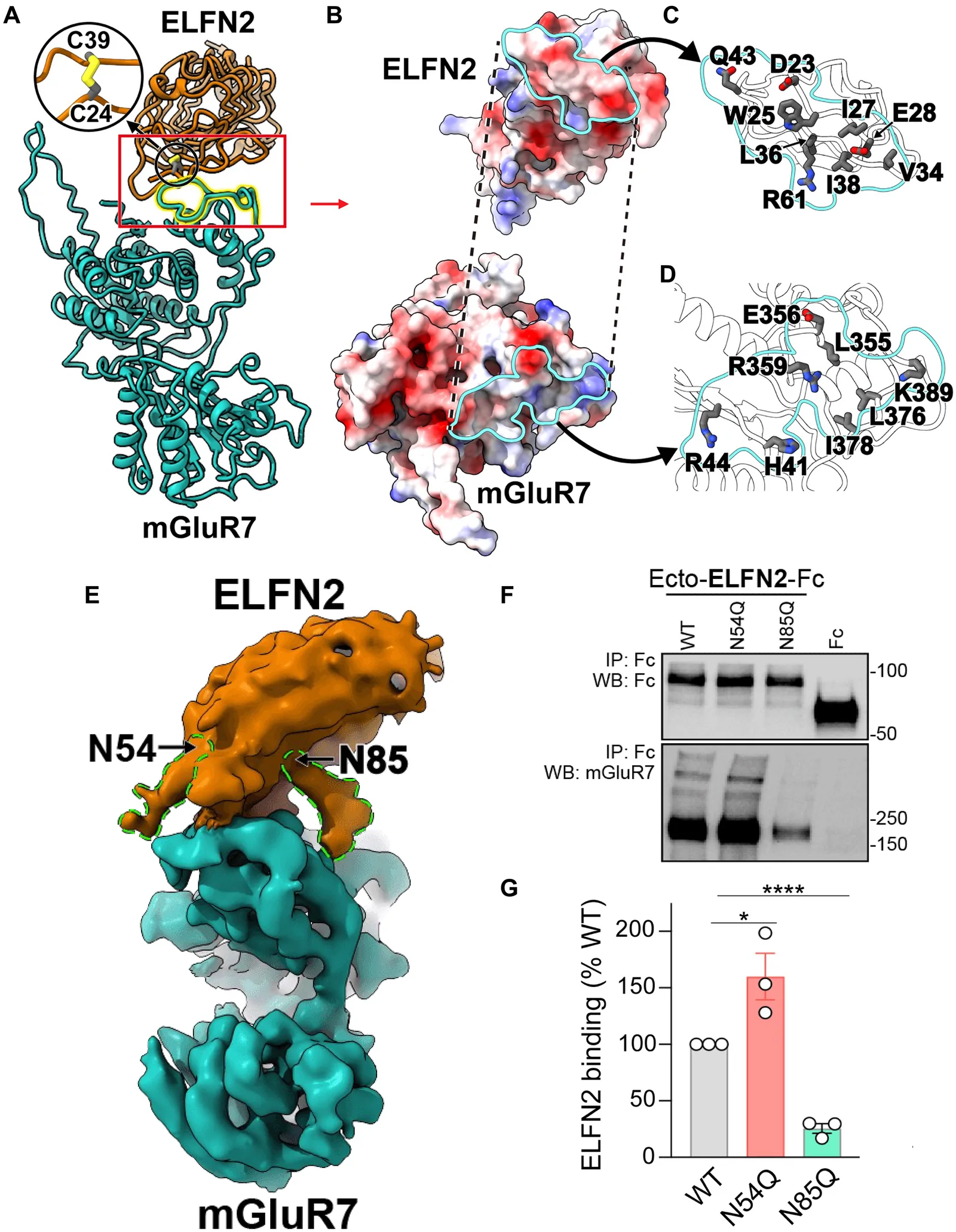
Organization of the mGluR7-ELFN2 interface. (**A**) The mGluR7-ELFN interface is composed of the region flanking helix αK and N terminus of mGluR7 and an N-terminal hairpin-like structure on ELFN2. Inset shows disulfide bond stabilizing ELFN2 hairpin-like structure. (**B**) Interacting residues exhibit complementary chemical surfaces of electrostatic and hydrophobic residues. Interacting residues are highlighted for (**C**) ELFN2 and (**D**) mGluR7. (**E**) Additional densities (arrow) present in cryo-EM reconstruction near binding interface correspond with N-linked glycosylation motifs on ELFN2. (**F**) Impact of mutating glycosylated residues in mGluR7 on interaction with Ecto-ELFN2 examined by coimmunoprecipitation followed by immunoblotting. This panel contains a subset of the dataset shown in fig. S6C. (**G**) Quantification of coimmunoprecipitation results in (F). Data are means ± SEM of three individual experiments. *P < 0.05 and ****P < 0.0001 (unpaired Student’s *t* test).

We have previously found that ELFNs contain a signal peptide that requires precise cleavage for mGluR binding: leaving aspartic acid as the first residue (D23 in ELFN2) in the mature protein (*4*). Our structural model revealed that the cleaved N terminus of the ELFN2 upstream of the LRR domain forms a hairpin structure stabilized by a disulfide at C24-C39 and fits into a groove on the mGluR7 VFT formed by helix αK and the linker regions immediately flanking it (Fig. 2A and fig. S2A). Overall, the interface buries ∼600 Å^2^ of solvent accessible surface area. The binding surfaces on mGluR7 and ELFN2 have complementary chemical landscapes (Fig. 2B). The core of the interaction is formed by a ridge of hydrophobic residues on ELFN2 comprising W25, I27, V34, L36, and I38 (Fig. 2C) that fit into a pocket formed by L355, I378, L376, and K389 side chains on mGluR7 (Fig. 2D), resulting in compatible surfaces with a hydrophobic hill in ELFN2 fitting into a hydrophobic valley in mGluR7. To probe the contribution of these individual residues to the overall binding interface, we created alanine substitution mutations of the key residues identified at the interface. ELFN2 mutations D23A, W25A, I27A, and E28A all expressed moderately well but lead to complete loss of binding with mGluR7 (fig. S2, B to D). The only exception was D23A, which bound to mGluR7 with wild-type (WT) affinity. On the other hand, mGluR7 mutations L355A and E356A had reduced expression (fig. S2, E and F). Whereas L355A lost any affinity for ELFN2, E356A bound with near WT affinity (fig. S2G), indicating that any ionic interactions that it engages in are not critical for ELFN2 binding. By contrast, mGluR7 mutations R359A, L376A, I378A, and K389A all expressed well but completely abolished ELFN2 binding.

Our reconstruction allowed us to dock the amino acid backbone chain into several regions that are disordered in previously reported structures of mGluR7 without ELFN bound. These include the linker region spanning T377-T386 between helices αK and αL (hereafter αK-αL loop), which makes multiple contacts with ELFN2 (Fig. 2A and fig. S2). Furthermore, several additional residues are resolved at the mGluR7 N terminus in ELFN2-bound structure. Last, the T137-V143 region on mGluR7 that participates in mGluR7 dimerization is much better resolved in our ELFN2-bound structure. The stabilizing influence of ELFN2 further indicates regions in mGluR7 involved in organizing the binding interface.

To further validate the network of residues involved in ELFN2-mGluR7 interactions, we used molecular dynamics (MD) simulation to characterize the conformational states accessible to the complex. Concatenated conformational states of both apo-mGluR7 and ELFN2-bound mGluR7 sampled across three independent replicates clustered into a dominant cluster, representing 35.3 and 36.3% of the apo and ELFN2-bound ensemble, respectively, indicating consistent convergence in both conditions. Alignment of the dominant cluster centroid from the ELFN2-bound simulation to our empirical model yielded a root mean square deviation (RMSD) of 4.527 Å (875 to 875 atoms), whereas the apo centroid showed a comparable RMSD of 4.815 Å (844 to 844 atoms), indicating that the global fold of mGluR7 is maintained regardless of ELFN2 presence. Despite this overall structural similarity, comparison of the apo and bound centroids reveals local conformational differences, particularly at the ELFN2 binding interface and mGluR7 homodimer interface (fig. S3). In particular, ELFN2 binding led to increased stability at the αK-αL loop (T377-T386) and dimerization interface loop (T137-V143). In the absence of ELFN2, the mGluR7 dimer interface became markedly reduced (fig. S4, A and B). Analysis of residue-residue contact frequencies over the final 500 ns of simulation showed that the ELFN2-mGluR7 interface recapitulated the core contacts observed in the cryo-EM structure (fig. S4, C to F). The hydrophobic core, in which ELFN2 residues W25, I27, V34, L36, and I38 engage a pocket formed by mGluR7 L355, L376, T377, I378, and K389, was broadly preserved in both protomers, with persistent contacts between ELFN2 W25/I27/V34 and mGluR7 L376/T377/I378 maintained throughout the simulations (fig. S5).

In the cryo-EM map, we observed additional densities protruding from ELFN2 that do not correspond to any amino acids and could represent glycosylated residues. These densities appear adjacent to predicted glycosylation motifs at N54, N80, N85, and N117 (Fig. 2D, and fig. S6, A and B) and (*26*). An additional motif at N205 is also predicted, but we could not observe additional mass in this region and thus cannot conclude whether it is glycosylated. In particular, the densities at N54 and N85 make direct contacts with mGluR7. To assess the contribution of these glycosylation sites in ELFN2 to mGluR7 binding, we mutated the asparagine residues to glutamine and measured the ability of soluble ecto-ELFN2 to coimmunoprecipitate with mGluR7. Notably, we observed a bidirectional effect. The ELFN2 N54Q glycosylation–incompetent mutant showed increased binding to mGluR7. In contrast, the N85Q mutation largely abolished mGluR7 interaction with ELFN2 (Fig. 2, F and G, and fig. S6C). Thus, glycosylation of ELFN2 substantially shapes its interaction with mGluR7.

ELFN2 shares very high sequence homology with ELFN1, which is particularly high (84%) in the N-terminal region between the first amino acid, D23, and the N85 glycosylation that mediates ELFN2 binding to mGluR7. We found that within this region, all the residues that participate in the organization of the mGluR7-ELFN2 interface are conserved (fig. S7, A and B). Conversely, docking the sequence of other group III mGluRs into mGluR7 further pointed to the conservation of the key residues that contribute to forming the binding interface with ELFN2 (fig. S7C).

**Fig. 3.**
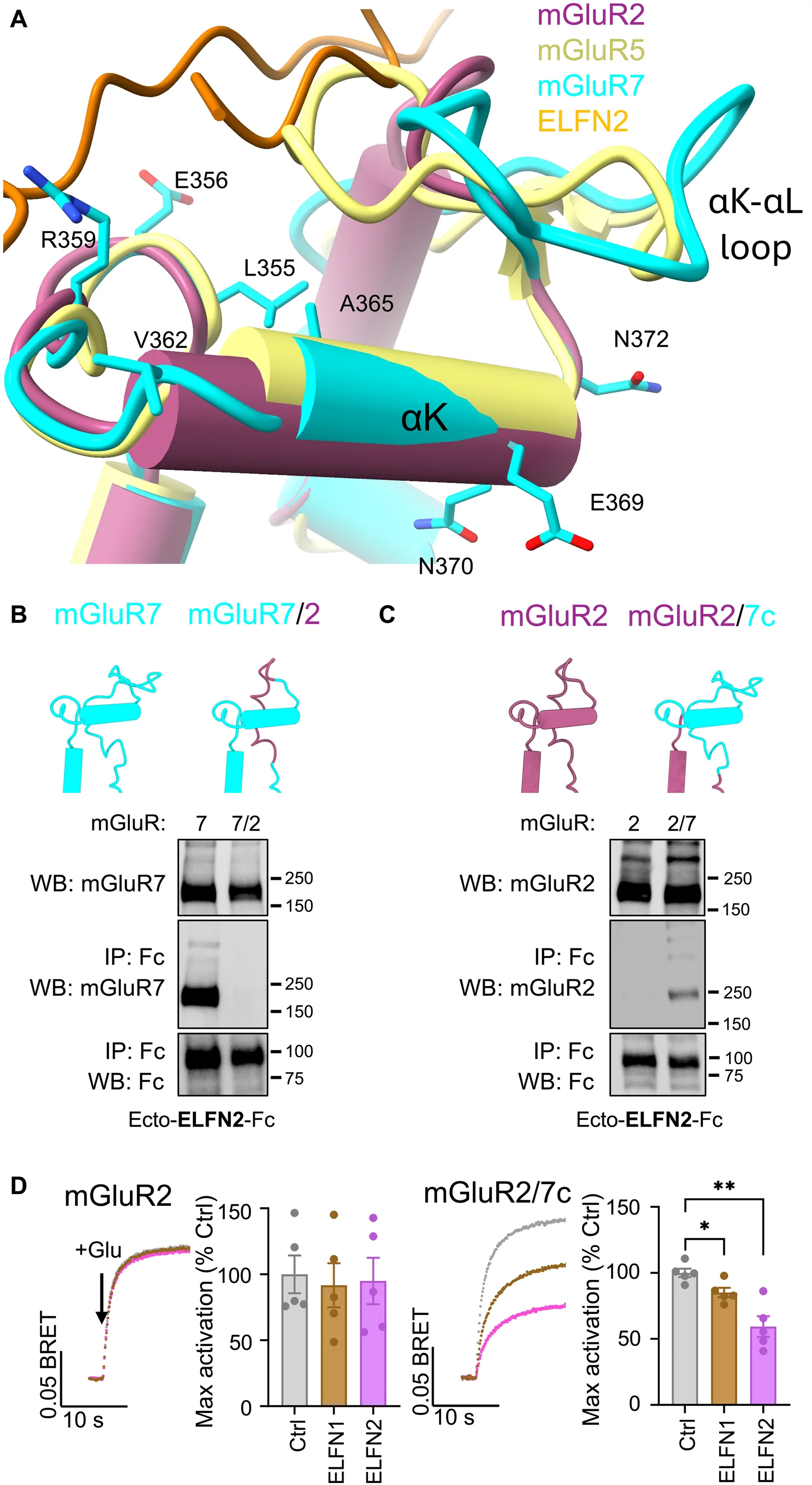
Selectivity determinants for group III mGluR interaction with ELFN proteins. (**A**) Overlay of mGluR structures demonstrating αK-αL loop is only compatible with ELFN binding in group III mGluRs. mGluR2 (PDB: 7EPA) and mGluR5 (PDB: 6 N52). The indicated residues are not conserved between group II and III mGluRs and are mutated in subsequent chimeras. (**B**) Chimeric strategy replacing αK-αL loop region (K375-Y402) from mGluR2 (magenta) onto mGluR7 (blue). Representative immunoblots of mGluR7 input (left), immunoprecipitation of ecto-ELFN2-Fc (bottom), and coimmunoprecipitation of mGluR7. This panel contains a subset of the dataset shown in fig. S8C. (**C**) Chimeric strategy placing ELFN-binding region (S356-F372) from mGluR7 (blue) onto mGluR2 (magenta). Representative immunoblots of mGluR2 input (left), immunoprecipitation of ecto-ELFN2-Fc (bottom), and coimmunoprecipitation of mGluR2. (**D**) Influence of ELFN proteins on Gαo activation by mGluR constructs as measured by the BRET assay. This panel contains a subset of the dataset shown in fig. S8F. Representative traces and quantification of maximal response amplitudes. Data are means ± SEM of five individual experiments. *P < 0.05 and **P < 0.01 (unpaired Student’s *t* test).

In the effort to understand the basis for ELFN-binding selectivity to group III mGluR, we compared the structures and sequences of all group III mGluRs, which bind ELFNs, and group I and II mGluRs, which do not bind ELFNs (Fig. 3A and fig. S8A) (*4*, *5*). We discovered that many of the contact residues, including mGluR7-L355, R359, L376, and K389, are conserved only in class III mGluRs but are not present in the corresponding regions of group I or II mGluRs (fig. S8A). Furthermore, the flexible αK-αL loop, which forms the main docking site for ELFN, adopts a distinctly different conformation in group I or II mGluRs, which is incompatible with ELFN binding (Fig. 3A). Last, distribution of charges and hydrophobics on the surface of group I and group II mGluRs is not complementary to the surface of ELFN1 and ELFN2 and is compatible only with group III mGluRs (fig. S7D).

To determine the necessity and sufficiency of these determinants in the selective recruitment of ELFN proteins, we generated a series of chimeras swapping key elements between mGluR7 and mGluR2, a group II mGluR that does not bind ELFN proteins (*4*, *5*). We started by replacing the mGluR7 αK-αL loop (K375-Y402), the key region involved in direct contacts with ELFN2 in mGluR7 for the corresponding loop from mGluR2. This manipulation completely abolished interaction with ELFN2 without affecting receptor expression (Fig. 3B). This chimera also lost binding to ELFN1, confirming necessity of the mGluR αK-αL loop for establishing interactions with both ELFN proteins (fig. S8B). We further tested the conservation with mGluR4, which yielded similar loss of both ELFN1 and ELFN2 binding upon replacing its αK-αL loop with that of mGluR2 (fig. S8C). Next, we turned to probing the sufficiency of the αK-αL loop for the mGluR interactions with the ELFN proteins, reciprocally grafting elements from mGluR7 into the respective regions on mGluR2 (fig. S8D). The same minimal replacement of mGluR2 S356-F372 of αK-αL loop in mGluR2 for the corresponding region from mGluR7 did not gain an ability to bind either ELFN1 or ELFN2 (fig. S8E). We then progressively enlarged the area to include more elements flanking the region including the αK helix itself. We found that the chimera with replacement of 48 amino acids (mGluR2/7c) gained the ability to bind both ELFN1 and ELFN2 (Fig. 3C and fig. S8E). Furthermore, coculture of this chimera with cells expressing ELFN1 or ELFN2 reduced the ability of this chimera to activate G proteins in response to glutamate in comparison to control cells (Fig. 3D and fig. S8F). This negative modulatory effect of ELFN mimics what has been observed for group III mGluRs, but not for other mGluRs, such as mGluR2 (*4*, *5*, *20*). These experiments show that the region in the vicinity of the mGluR αK-αL loop is both necessary and sufficient for both ELFN binding and the regulatory allosteric effects that this binding imparts on mGluRs. We further tested the effect of the soluble extracellular domain of ELFN2 (ecto-ELFN2). We observed that this soluble ELFN2 also reduced the ability of mGluR4, but not that of mGluR2, to activate G proteins, as observed with the full-length transmembrane protein (fig. S8G). This further argues that ELFN action involves allosteric regulation of mGluRs as opposed to merely interfering with receptor dynamics due to membrane tethering.

### An allosteric network connects ELFN-binding site to orthosteric pocket

**Fig. 4.**
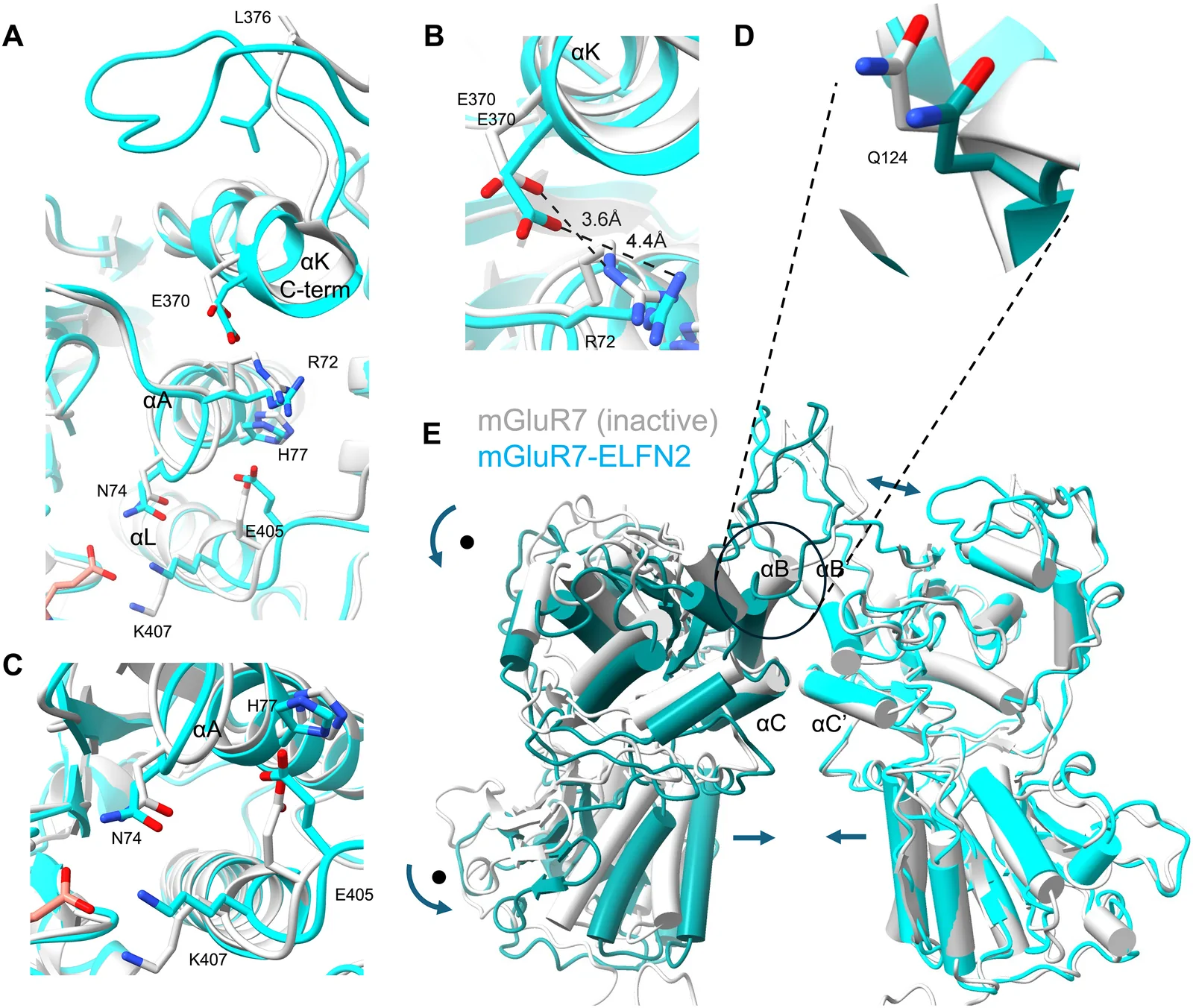
An allosteric network of residues connecting ELFN-binding site to glutamate binding site. (**A**) Overview of mGluR7 residues involved in allosteric network activated by ELFN binding. Residues in the ELFN2-bound state are in blue, and residues in the unbound state are in light gray (PDB: 7EPC). (**B**) Restructuring of helix αK disrupts salt bridge between E370 and R72 on helix αA. (**C**) Conformational changes propagate to alter interaction between H77 and E405. This causes a restructuring of residues N74 and K407 that interact with glutamate (pink). (**D**) Displacement of Q124 on helix αB. (**E**) Disruption of mGluR homodimer interface and changes in relative orientation of each mGluR7 VFT subunits induced by ELFN binding.

Our structure provides an opportunity to understand the mechanisms of allosteric regulation of group III mGluR activity by ELFN proteins. To obtain insights into the structural basis underpinning this regulation, we compared our structure of ELFN2-bound mGluR7 with inactive, apo-mGluR7 (PDB: 7EPC). We observed many notable changes. Starting at the ELFN-binding interface, mGluR7 L376 on the αK-αL linker is pushed downward to accommodate interaction with ELFN2 (Fig. 4A). This rearrangement of the αK-αL linker causes an unraveling of the C terminus of helix K. In the unbound state, these residues, Y367-N371, are in a π-helical arrangement. It appears that this arrangement is partially stabilized through an interaction between the side chains of R72 on helix A and E370 on helix K (Fig. 4B): a region necessary for the induction of ELFN binding on mGluR2 chimeras (Fig. 3, C and D; and fig. 8, E and F). Additionally, an ionic interaction between H77 and E405 is disrupted causing the nearby K407 residue to shift (Fig. 4C). In structures of active mGluRs, this residue interacts with glutamate and other agonists. Thus, we can trace an allosteric network of residues from the ELFN2 interaction site down to disruptions in the glutamate binding pocket.

We further observed that ELFN2 binding causes rearrangement of each mGluR7 protomers relative to each other. The VFT domains contact primarily at the helix αB-αC homodimer interface. With ELFN2 bound, the distance between dimers increases by ∼3 Å at Q124 on αB (Fig. 4D). Canonically, mGluR activation involves closure of the VFT domain and a decreased intersubunit helix αB-αC angle. These two changes result in the C-terminal lobes of the VFT domain traveling 14 Å into closer proximity, allowing the CRD and 7TM to rearrange into the active conformation (*27*). In the ELFN2-bound structure, we see the C-terminal VFT lobes moving 6.3 Å closer together at S237, resembling partial activation (Fig. 4E). Intriguingly, we do not observe any rotation at the helix αB-αC homodimer interface, and the VFT only closes very slightly. Rather, the approximation of the C-terminal VFT lobes is due to an overall rotation of each VFT homodimer into the plane of the homodimer interface. Thus, ELFN binding appears to produce a distinct pattern of changes in multiple networks of residues that connect the allosteric site and VFT, thereby affecting the conformational state and resulting activity of mGluRs.

To evaluate the conformational dynamics of our structure, we applied 3D variability analysis (*28*) to our particles. This analysis revealed additional classes: an apo-mGluR7 class with no ELFN2 bound; a class with one ELFN2 monomer bound; a class with two ELFN2 monomers bound, but no change in the position of the VFT and stalk domains (inactive); and lastly the class described above with rotation of the VFT domains and approximation of the stalk domains (partially active) (movie S1). While the resolution of these classes was not sufficient to build models, we were able to observe notable differences between the classes supporting the notion that ELFN2 binding induces the conformational changes identified above.

### Cooperativity drives enhanced mGluR association with ELFN upon activation

**Fig. 5.**
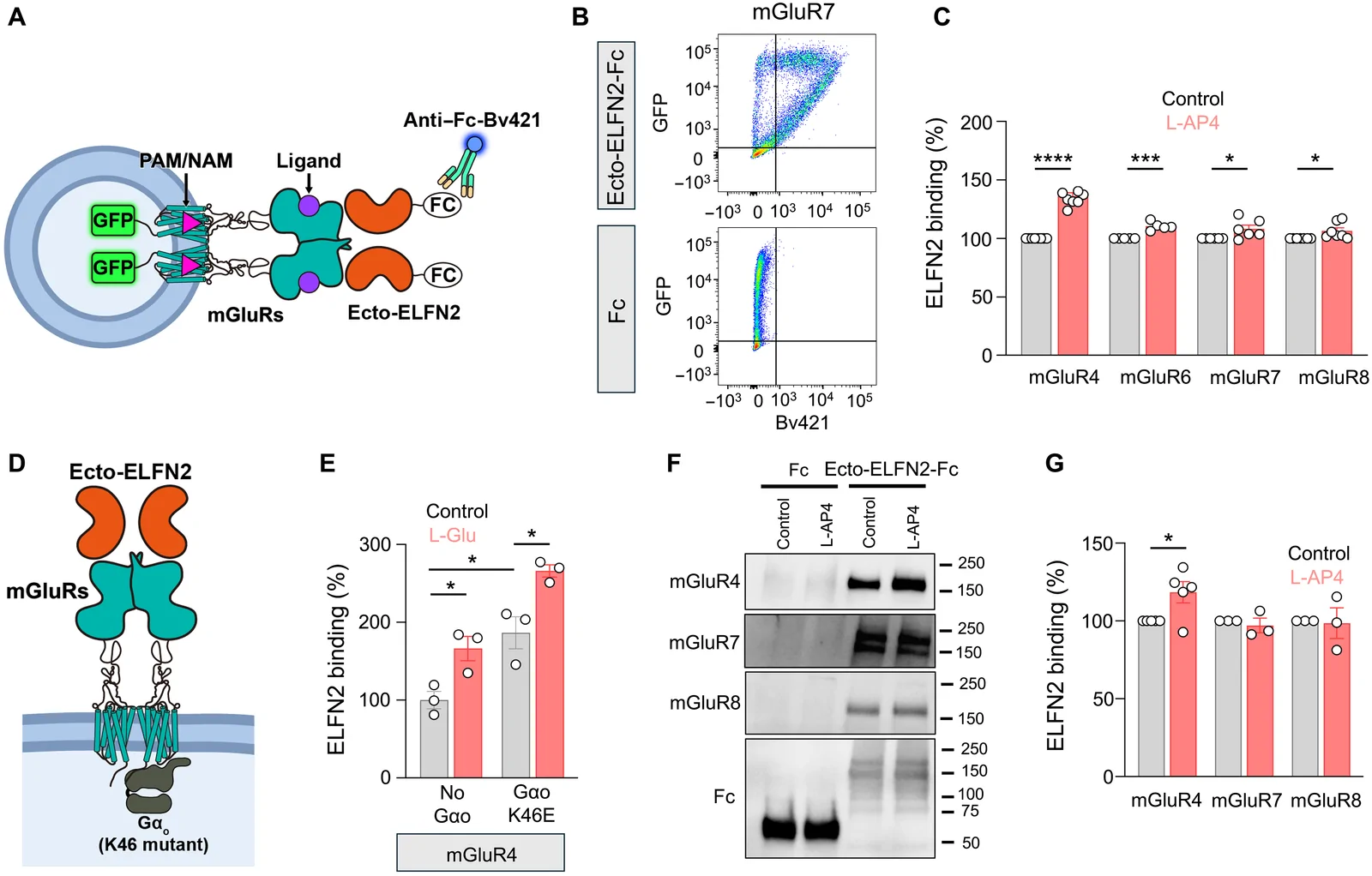
mGluR activation increases binding affinity for ELFN. (**A**) Schematic of cell sorting assay for measuring ELFN binding to mGluR. Green fluorescent protein (GFP)–tagged mGluR–expressing cells are treated with either soluble ecto-ELFN-Fc or Fc control proteins in the presence of anti–Fc-Bv421 antibodies. Cells are further exposed to mGluR ligands to assess their effects on mGluR-ELFN binding. (**B**) Representative flow diagrams sorting cells based on GFP and Bv421 fluorescence. (**C**) The effect of agonist L-AP4 on median fluorescence intensity ELFN2 signal for each group III mGluR. Data are means ± SEM of five to eight individual experiments of three replicates each (unpaired Student’s *t* test). (**D**) Gαo K46E mutant binding has cooperative effect on mGluR-ELFN affinity. (**E**) The effects of glutamate and Gαo K46E expression on mGluR4 interaction with ELFN2. Data are means ± SEM of three experiments [two-way analysis of variance (ANOVA) with Tukey’s post hoc test]. (**F**) Representative immunoblots and (**G**) their quantification examining pull-down of mGluRs in synaptosome preparation by recombinant ecto-ELFN2-Fc. Data are means ± SEM of three to five individual experiments. *P < 0.05, ***P < 0.001, and ****P < 0.0001 (unpaired Student’s *t* test).

The existence of the allosteric network connecting the ELFN-binding site in mGluRs with the orthosteric binding pocket raised an intriguing possibility that the activation of the group III mGluRs may reciprocally influence its interaction with ELFN proteins in a cooperative (i.e., reverse allosteric) mechanism. To test this hypothesis, we devised a flow cytometry assay to measure binding of soluble ELFN ectodomain to full-length mGluR expressed on the cellular surface in the absence or presence of a ligand (Fig. 5, A and B). First, we systematically characterized interactions with ELFN1 and ELFN2 across all members of group III mGluR (fig. S9). At the basal state, in the absence of a ligand, ELFN2 bound all group III mGluRs with greater affinity than ELFN1. Both ELFN proteins had similar binding affinity for mGluR6, mGluR7, and mGluR8, but their binding to mGluR4 had markedly (∼10-fold) lower affinity. Notably, we found that the addition of orthosteric agonist L-AP4 increased ELFN-binding affinity to all group III mGluRs (Fig. 5C). In contrast, the addition of mGluR orthosteric antagonist CPPG had no effect on ELFN binding (fig. S10A). Of all group III mGluRs, the greatest effect of agonist simulation on both ELFN1 and ELFN2 binding was observed for mGluR4, possibly due to its lowest binding affinity for ELFN proteins under unstimulated conditions, leaving a larger dynamic window (fig. S 10, B and C). Additionally, similar effects on ELFN binding were observed when stimulated with either glutamate or L-AP4. The negative modulatory effect of ELFN was previously shown to be indifferent to which agonist caused stimulation (*4*). To further probe the relationship between the activation status of mGluR, its conformational states, and binding to ELFN proteins, we extended these studies to test various allosteric modulators available for mGluR4 (*29*–*38*) and mGluR7 (*38*–*41*) with distinct binding sites and mechanisms. We found specificity in their effects with several mGluR4 PAMs increasing ELFN binding: i.e., ADX88178 (*29*), Valiglurax (*33*), and VU0361737 (*32*) (fig. S10D), with others, i.e., mGluR7 PAM AMN082 (*39*) and VU0422288 (*38*), mGluR7 inverse agonist MMPIP (*40*), and mGluR7 NAM XAP044 (*41*), showing no effects (fig. S10E). These pharmacological studies indicate that the conformational changes upon mGluR activation enhance their association with ELFN proteins. We also tested whether adding glutamate allowed synergy with PAMs to increase ELFN affinity, but this was only observed for PAMs that already increased ELFN affinity without glutamate stimulation (fig. S10F).

In our structural determination, we included the compound MMPIP to stabilize the inactive state of mGluR7. As ELFN2 appears to increase mGluR7 activity in the absence of agonist, we questioned whether this behavior persisted even in the presence of MMPIP. We cocultured mGluR7-expressing cells with ELFN2-expressing or control cells. We found that coculture with ELFN2 resulted in increased levels of G protein activation under nonstimulated conditions, consistent with ELFN2 increasing mGluR7 basal activity (fig. S10G). After addition of MMPIP, we likewise observed a significant increase in G protein activation in the presence of ELFN2 (fig. S10H). This indicates that the activating properties of ELFN2 under basal conditions remain intact even when MMPIP is present.

Next, we probed the contribution of G proteins to the cooperativity. G protein binding is well-known to produce allosteric effects on GPCRs by increasing their affinity for orthosteric ligands (*42*–*45*). To stabilize the mGluR in the G protein–bound state, we took advantage of a newly developed reagent, the K46E Gαo mutant that constitutively binds to GPCRs in a nucleotide free state (*45*). Notably, expression of K46E Gαo selectively enhanced binding of ELFN2 to mGluR4 (Fig. 5, D and E), but not other group III receptors, both in the absence and presence of an agonist (fig. S11). Glutamate agonism further potentiated the effect of K46E Gαo suggesting that both orthosteric agonists and G protein binding cooperate to alter ELFN association dynamics.

To explore the relevance of the observed cooperativity effects in the endogenous synaptic setting, we have analyzed ELFN interactions with mGluRs in the brain synaptosomal preparations. We found that stimulation with L-AP4 increased the binding of ecto-ELFN2 to endogenous mGluR4, but not other group III mGluRs (Fig. 5, F and G). These observations suggest that increased glutamatergic signaling enhance stabilization of mGluR4 to synapses through transsynaptic interactions with ELFN proteins. Mechanistically, these observations further suggest that the signaling status of mGluRs can be transmitted inside out to regulate the apical ELFN-binding site in a cooperative manner.

### ELFN prevents group III mGluRs from reaching the fully resting and active states

**Fig. 6.**
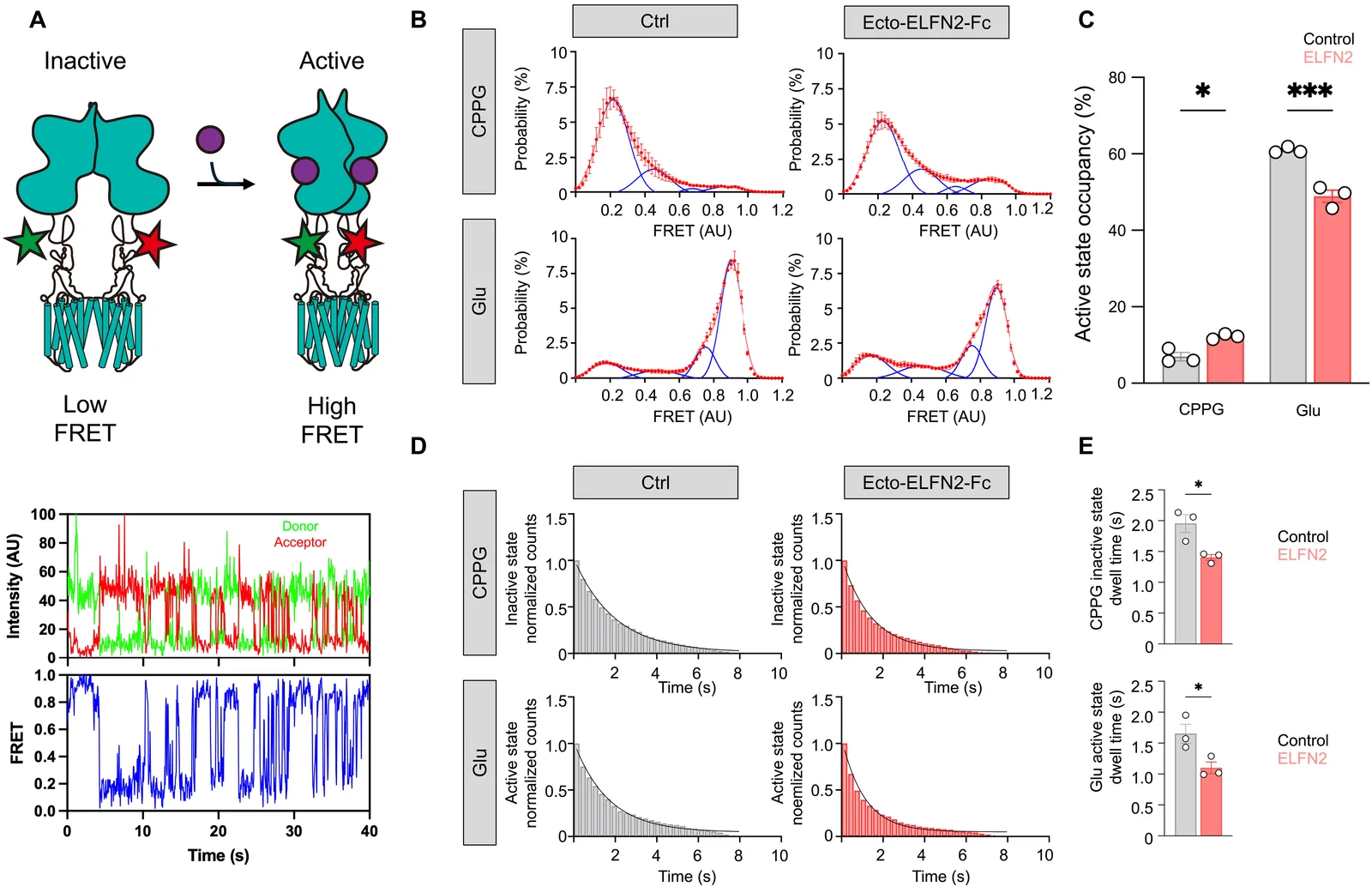
ELFN binding shifts mGluR into an intermediate activation state. (**A**) Location of FRET donor and acceptor probes on mGluR4 with representative smFRET traces. Donor, acceptor, and FRET signals are in green, red, and blue, respectively. (**B**) smFRET population histograms of mGluR4 FRET sensor in the presence of 0 (with 1 mM CPPG) or 1 mM glutamate and 0 or 0.6 μM ecto-ELFN2. The idealized fit (red) is based on 4 Gaussian distributions (blue) representing each conformational state. Peaks are centered around 0.20, 0.50, 0.70, and 0.87 FRET, indicative of inactive, low intermediate, high intermediate, and active states, respectively. In the absence of glutamate, ecto-ELFN2 alters intermediate-FRET state occupancy (0.5 and 0.70 FRET) and slightly increases the active high-FRET state. In saturating glutamate, ecto-ELFN2 primarily reduces the active high-FRET state. Data are means ± SEM of three individual experiments. (**C**) Active high-FRET state occupancy is shown as a function of glutamate and ecto-ELFN2 in the graph and is calculated as area under the curve (two-way ANOVA with Tukey’s post hoc test) (**D**) Low-FRET state (inactive) dwell-time histogram for CPPG-treated samples (top) and high-FRET state (active) for dwell-time histograms for glutamate treatment (bottom) with and without ecto-ELFN2. Data fit to a single exponential decay (black curve) and (**E**) quantified (unpaired Student’s *t* test). Data are means ± SEM of three individual experiments. *P < 0.05, ***P < 0.001.

To further dissect the mechanisms by which ELFN binding alters group III mGluR function, we used single-molecule fluorescent resonance energy transfer (smFRET) experiments to directly measure and quantify dynamic changes in mGluR conformations induced by ELFN binding. Given the highly flexible nature of mGluRs, smFRET experiments have been valuable in interrogating the distributions of mGluR conformations and activation dynamics under various conditions (*46*–*49*). In the present study, we focused on the mGluR4-ELFN2 interaction because of the high response in affinity of their interaction to glutamate stimulation. To achieve high resolution in our analysis, we focused on the CRD of mGluR4 that undergoes a large range of conformational changes during the activation. We used unnatural amino acid substitution to site-specifically label the CRD of mGluR4 as described previously (Fig. 6A and fig. S12A) (*48*). In the absence of glutamate and presence of mGluR4 antagonist CPPG, we observed that addition of ecto-ELFN2 decreased the occupancy of the low-FRET state corresponding to the inactive receptor state (fig. S12B) and increased the occupancy of the FRET state corresponding to the fully active conformation of mGluR4 (Fig. 6, B and C). Next, in the presence of a saturating concentration of glutamate, we saw a significant increase in the occupancy of the active state, as expected. However, in this case, addition of the ecto-ELFN2 reduced the occupancy of the active state. Dwell-time analysis shows that the increase in the occupancy of the active state is partially due to changes in the lifetime of the active state, which is dependent on both the presence of ELFN2 and the orthosteric ligand (Fig. 6, D and E). Overall, these results indicate that ELFN binding exerts dual effects on mGluR4, promoting its shift to the active state in the absence of receptor activation and, at the same time, inhibiting it from reaching the fully active state when the receptor is activated by orthosteric ligands.

The reduced stability of the mGluR4 active state suggests that ELFN2 binding is not compatible with mGluR activation. Mapping the ELFN2-mGluR7 interface on top of active mGluR4 (PDB: 7E9P) allowed us to create a homology model of the active complex (fig. S13A). Overall, the ELFN-binding site remains sterically accessible between the ligand-free ELFN2-mGluR7 and active mGluR4 structures (fig. S13B). Intriguingly, to maintain binding, the ELFN2 LRR domains must rotate away from each other and the distance between LRR domains increases. ELFN proteins are believed to form homodimer contacts (*50*), but this orientation and separation of ELFN monomers precludes dimerization within the protomer, at least near the LRR domains. It is possible, however, that mGluRs form higher-order structures of multiple dimers with ELFN forming dimer contacts between protomers in these higher-order structures (fig. S13C). It is possible that that agonist-induced activation of mGluRs may interfere with ELFN dimer interfaces as well. However, the exact residues involved in ELFN dimerization remain unknown.

### Disruptions in mGluR-ELFN complex span several NDDs

**Fig. 7.**
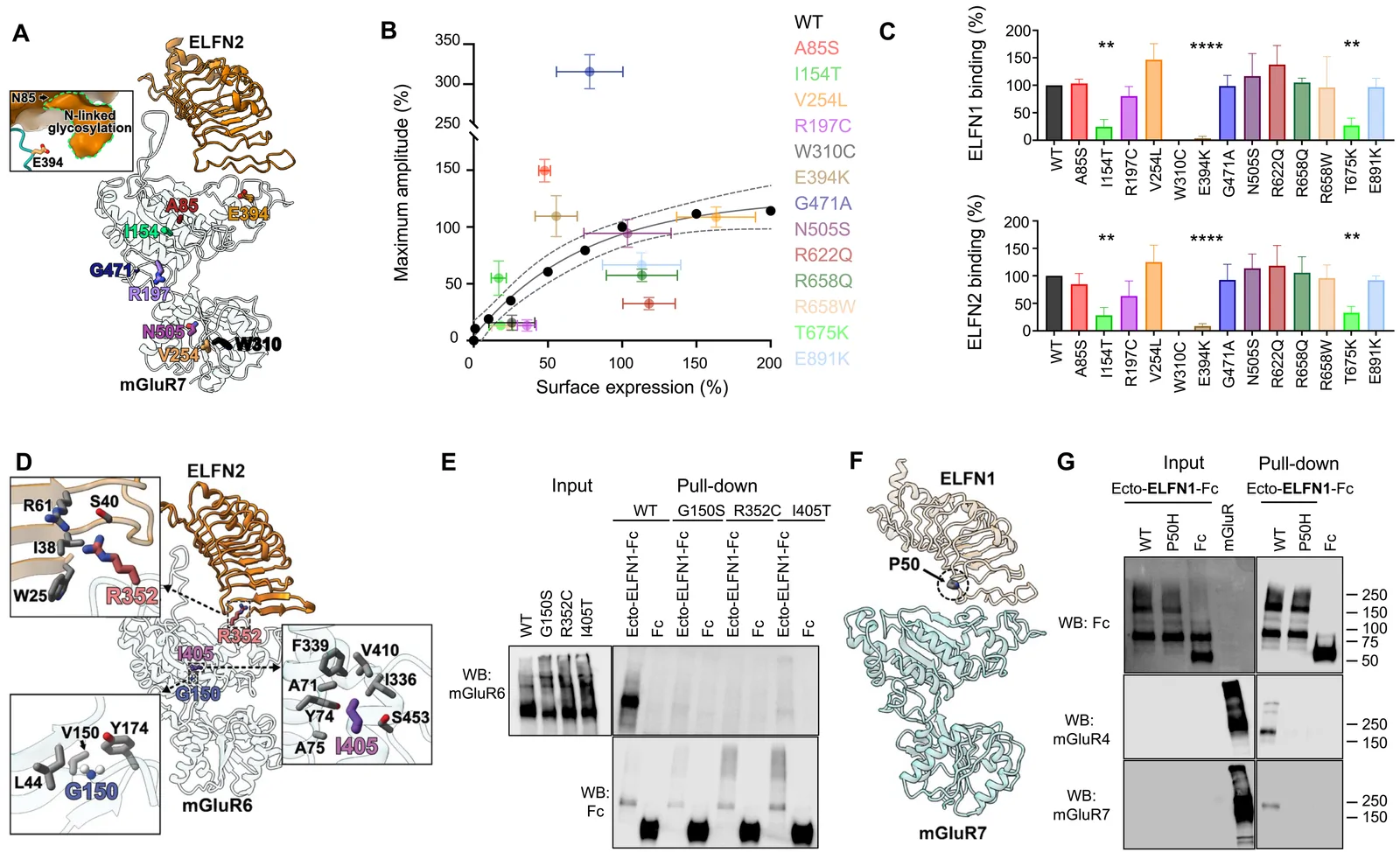
Disruption of the mGluR-ELFN interaction is a recurring disease mechanism. (**A**) Location of pathogenic mutations on mGluR7 structure. (**B**) Scatter plot of BRET-based Gαo activation sensor versus surface expression of each mutant. (**C**) Normalized binding of each mGluR7 mutant to ELFN1 and ELFN2 based on immunoblots of immunoprecipitated ecto-ELFN1/2-Fc proteins. (**D**) Location of pathogenic mGluR6 mutations near ELFN-binding interface and related allosteric networks. (**E**) Representative immunoblots of WT and mutant mGluR6 input (left), immunoprecipitation of ecto-ELFN1-Fc (bottom), and coimmunoprecipitation with mGluR6. This panel contains a subset of the dataset shown in fig. S14F. (**F**) Location of ELFN1 P50H in relation to mGluR7 binding interface. (**G**) Representative immunoblots of WT and mutant mGluR6 input (left), immunoprecipitation of ecto-ELFN1-Fc (bottom), and coimmunoprecipitation with mGluR6. Data are means ± SEM of three to four individual experiments. **P < 0.01 and ****P < 0.0001 (unpaired Student’s *t* test).

The structure of the mGluR7-ELFN2 complex offers an opportunity to provide insights into the mechanisms underlying NDDs caused by mutations in mGluR7. To this end, we studied a comprehensive panel of 14 clinical mGluR7 variants (*14*), examining their expression, localization, functional activity, and interaction with ELFN proteins, because the molecular mechanisms have not been established for any variants implicated in this disease (Fig. 7A). While most of the mutants were readily expressed in human embryonic kidney (HEK) 293 cells, only approximately half of them showed unperturbed localization on the cell surface (fig. S14, A and B). We next analyzed their functional activity by using a bioluminescence resonance energy transfer (BRET)–based assay that monitors the ability of GPCRs to activate the G proteins (fig. S14C). To differentiate mutants whose apparent loss in G protein signaling was driven by deficits in plasma membrane localization, we correlated the activity data with the surface expression in reference to the behavior of the WT mGluR7 (Fig. 7B). We found that, when we examine the activity of the mGluR7 mutants, an interesting pattern emerged. Several mGluR7 mutants exhibited greater response to glutamate at the equivalent to WT levels at the surface (Fig. 7B). All these mutations (A85S, I154T, E394K, and G471A) are located in the N-lobe of mGluR7. Mutations in the 7TM region (R622Q, R658Q, R658W, T675K, and E891K) all lead to decreased glutamate response. Last, we analyzed the impact of the mutations on ELFN binding. Pull-down assays performed with the whole-cell lysates revealed that four mutations (I154T, W310C, E394K, and T675K) markedly affect the binding of mGluR7 to recombinant Ecto-ELFN1 or Ecto-ELFN2 (Fig. 7C and fig. S14D). Of these four, only E394K exhibited moderate levels of surface expression (fig. S14, A and E). Thus, I154T, W310C, and T675K are improperly trafficked and may be missing important stabilizing posttranslational modifications that allow them to bind ELFNs. By contrast, E394K traffics moderately well, but is still incapable of binding ELFNs. Notably, E394 is the only residue located directly at the interface with ELFN2 and is predicted to be involved in the interaction with the N85-linked glycosylation in ELFN2 that plays an essential role in binding to mGluR7.

To understand whether the disruption in ELFN interaction could contribute to a disease, we next focused on pathogenic mutations in mGluR6, which lead to congenital stationary blindness. Our analysis indicate that, among ∼22 presently known pathogenic mutations (*51*–*63*), three (G150S, R352C, and I405T) map in the ELFN-binding interface or near the ELFN allosteric networks and could be predicted to affect the binding (Fig. 7D). R352 is the paralogous residue to R359 in mGluR7, which interacts with W25 on ELFN2. G150 and I405 both reside within the core of the N-lobe of the VFT. I405 sits on helix αL and is peripheral to several residues in the ELFN allosteric network. It is downstream of the paralogous mGluR7 K405 that interacts with glutamate. It also contacts hydrophobic A71 and A75 on helix αA. The I405T mutation likely interferes with the hydrophobic packing of these two helices. To test these predictions, we expressed the three mutants and found that they could not bind soluble ecto-ELFN1 and ecto-ELFN2 (Fig. 7E and fig. S14F).

Last, we explored the mutations in ELFN1 that have been also recently reported to cause profound developmental and intellectual impairment. Symptoms can include epilepsy, autistic like features, and dysmorphia with substantial phenotypic resemblance to mGluR7 disorder. Of the four mutations described to date (*17*, *18*), only one is a missense variant that leads to a single–amino acid mutation: P50H. Our analysis indicates that it is positioned at the binding interface with mGluR7. We predicted that alteration of the specific backbone geometries of proline at this position will likely substantially alter the geometry of the mGluR-ELFN–binding interface (Fig. 7F). We tested this prediction by studying the binding of recombinant ecto-ELFN1 with P50H mutation and found that it completely lost the ability to bind to mGluR7 and mGluR4 (Fig. 7G). The P50H mutation also completely abolished binding of full-length ELFN1 to mGluR4, mGluR7, and mGluR8 (fig. S15). Together, these results indicate that disruption in the complexing between group III mGluR and ELFN proteins is a recurring mechanism underlying the cause of NDDs.

## DISCUSSION

This study provides mechanistic insights on how cell adhesion proteins of the ELFN class engage and regulate group III mGluRs. ELFN proteins are well established for the fundamental role that they play in regulating neurotransmission and short-term plasticity of excitatory synapses in the nervous system (*3*, *5*–*7*, *19*, *21*). The key to their function is the ability to transsynaptically bind mGluRs, stabilizing them proteolytically and optimally positioning them for synaptic regulation. Recent work further demonstrated that ELFNs also serve to allosterically regulate mGluRs, enhancing their basal activity but diminishing their responsiveness to glutamate (*4*). The structure of the mGluR7-ELFN2 complex and the detailed functional investigation that we describe here explain how ELFN proteins regulate mGluR activity.

We found that group III mGluRs and ELFNs interact with nanomolar affinity through a conserved interface of hydrophobic and electrostatic interactions. We further uncovered how this interaction is regulated by distinct N-linked glycosylation sites on ELFN that play crucial role in shaping the interaction in a bidirectional manner. Our data suggest that ELFN2 binding has a stabilizing effect on the intrinsically flexible regions at the apical surface of the mGluRs likely explaining the protective effect from proteolytic degradation (*4*, *19*, *20*). The N termini of mGluR7 are located near the ELFN-binding interface, together with the N termini of ELFN2, the helix αK-αL loop, and the N54-linked glycosylation site of ELFN2. Prior studies determined that any extension of the N terminus of ELFN proteins abolish their interaction with mGluRs (*4*). Similarly, we found that the glycosylation at the N54 site is also detrimental for binding and its removal enhances mGluR-ELFN2 interaction. In contrast, we found that removal of the adjacent N85-linked glycosylation from ELFN2 greatly diminished its interaction with mGluRs. These observations indicate that the strength of ELFN-mGluR interaction could be regulated by posttranslational modifications that alter the N termini or glycosylation status of ELFN proteins.

Previous studies reported that mGluR7 interaction with ELFN1 partially depended on the two mGluR7 glycosylation sites, N486 and N572 (*26*). Our structure shows that both of these residues are located far from the mGluR-ELFN interface and, therefore, are not likely to engage in direct interactions. Furthermore, mGluR6 were also reported to contains glycosylation sites necessary for ELFN binding, which do not completely correspond with mGluR7 (*64*). However, these residues are also located away from the binding interface based on our structure. Because these glycosylation sites were also found to be important for the localization of mGluRs on the surface and at the synapses, we think that the changes in trafficking caused by mutation of the corresponding residues could have aberrantly altered the posttranslational processing of these mGluRs and likely confounded the interpretation of these findings.

**Fig. 8.**
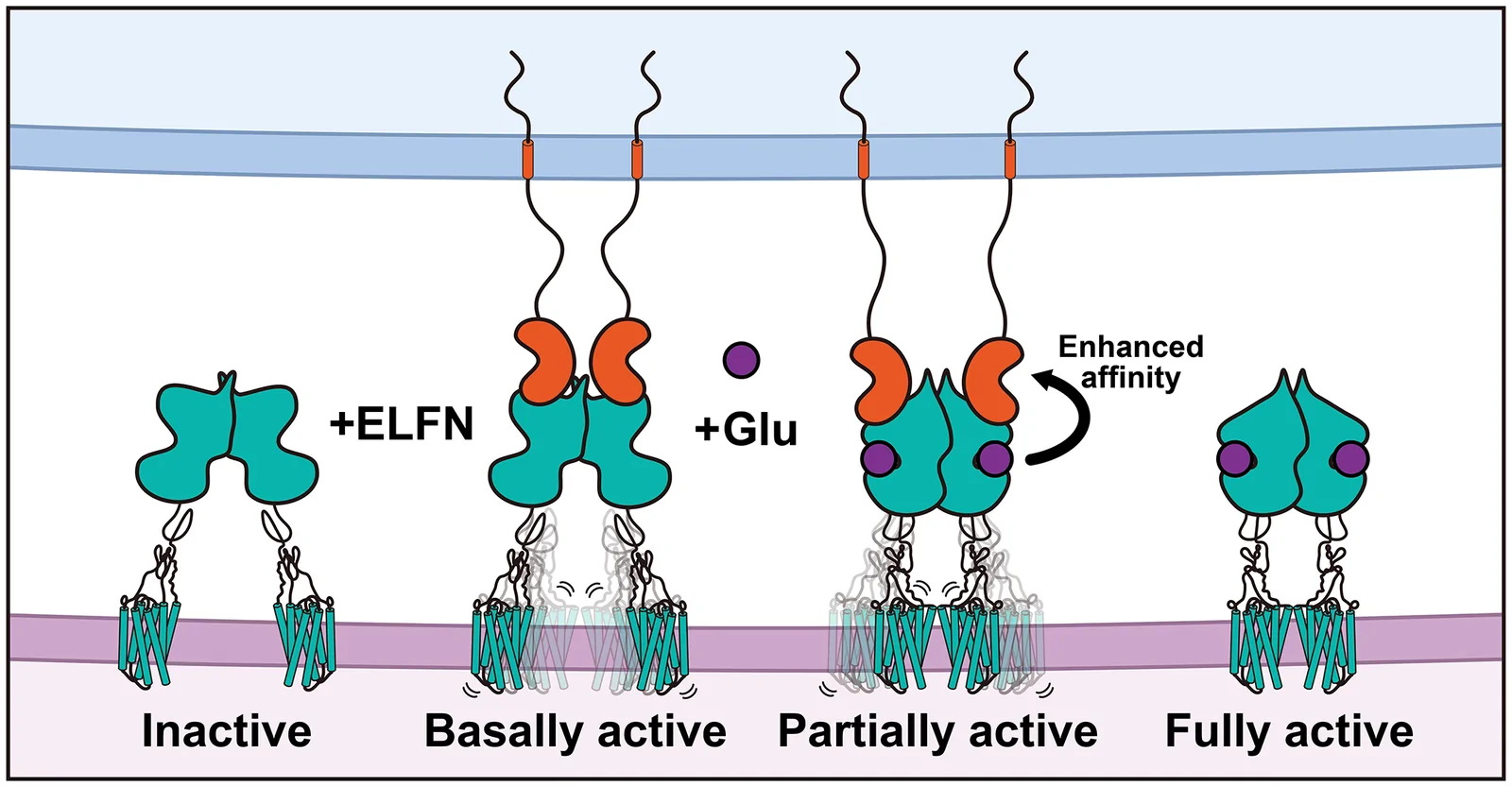
Model of ELFN-mediated regulation of group III mGluRs. In the absence of ELFN or glutamate, group III mGluRs are stabilized in the inactive state. Binding of ELFN increases constitutive activity and moves mGluRs out of an inactive state into a basally active state. ELFN binding also stabilizes group III mGluR at the synapse. Binding of glutamate enhances the affinity of group III mGluRs for ELFN, but ELFN keeps the mGluR in an only partially active state. When ELFN is not bound, group III mGluRs can enter the fully active state.

In this work, we define the molecular basis for the allosteric influence of ELFN on mGluR activity. We document that ELFN binding produces coordinated shifts in the conformation of the mGluR7 dimeric interface and a network of residues that connect the docking site for ELFN with the orthosteric ligand binding pocket. We also observe disruption of the glutamate binding pocket with ELFN2 bound, consistent with decreased glutamate sensitivity (*4*, *5*, *20*). These structural observations are consistent with the smFRET measurements where addition of ELFN blunted the proportion of mGluR4 in the active state in response to glutamate. These effects likely explain the negative allosteric effects of ELFN binding on mGluR agonist–dependent signaling (*4*, *5*, *20*). Our structure-functional studies help further explain the positive allostery induced by ELFN binding on the ground state of mGluRs in the absence of the agonist. We observe that ELFN interaction induces a movement in the VFT C-terminal lobes that resembles partial activation. Again, these changes are consistent with the smFRET measurements where addition of ELFN increased the fraction of mGluR in the fully active conformation. Together, these observations suggest a model where ELFN pushes mGluR out of the resting state and into an intermediate state but destabilizes mGluR in the active state (Fig. 8). This pharmacological behavior categorizes ELFN proteins in the rare class of agonist-negative allosteric modulators. This is especially unusual considering that ELFN is a protein. It is intriguing to compare these observations to recent findings on modulation of mGluR5 by a nanobody (Nb43) that acts as a positive allosteric modulator (*27*). Intriguingly, Nb43 binds in the same groove on the VFT domain as ELFN2. Although the specific binding determinants differ, this region of mGluRs likely represents a critical allosteric control site and could serve as a promising target for synthetic binders.

We also note that a recent structure of apo-mGluR7 in native membrane vesicles (PDB: 9OMO) (*65*) is virtually indistinguishable from the same structure in detergent [PDB: 7EPC (*23*); RMSD, 0.351 Å]. Because the lipid environment of the native membrane micelles likely resembles the environment in our detergent-free native nanodiscs, this argues against the notion that lipid membrane composition could be responsible for the changes we describe. Additionally, our structure uses the compound MMPIP to stabilize the inactive state of mGluR7, as has been used previously (*23*). Nevertheless, MMPIP does not alter the structure of mGluR7 (*65*). Additionally, MMPIP does not interfere with ELFN2 binding (fig. S10E) or activity (fig. S10H).

One of the key findings reported in this study is the observation of the cooperative influence wherein the activation status of mGluRs tunes their interaction with ELFN, especially mGluR4. The facts that mGluR4 PAMs also increase affinity for ELFNs and orthosteric antagonists do not indicate that the cooperativity is coincident with orthosteric ligand binding, but such binding is not strictly necessary. Rather, ELFN-binding affinity is increased by active mGluR conformations and not necessarily the specific conformations of residues in the glutamate binding pocket. We think that this cooperative regulation has a biological significance in that it would lead to increased transsynaptic synaptic recruitment of autoinhibitory mGluRs upon increases in the glutamatergic signaling, thereby serving as a homeostatic scaling mechanism. The differences in the cooperative effects between mGluR4 and mGluR7 are notable. Among class III mGluRs, mGluR7 has the lowest sensitivity to glutamate and, by itself, may not even respond to endogenous levels of glutamate (*66*, *67*). Rather, it has been noted that mGluR7 exhibits high levels of constitutive activity (*6*, *68*). As ELFN can modulate mGluR7 constitutive activity (*4*, *6*), ELFN likely plays an important role in regulating mGluR7 activity apart from endogenous glutamate. On the other hand, mGluR4 robustly activates G proteins in response to glutamate. It is then reasonable that mGluR4 would also exhibit a greater degree of cooperativity in response to agonist.

Overall, the data described here provide a unified model of dynamic mGluR tuning by ELFN proteins. During synaptogenesis, ELFNs transsynaptically bind and stabilize class III mGluRs at glutamatergic synapses. Then, under basal conditions, ELFNs increase the basal activity of mGluRs and prevent unprovoked synaptic discharge, reducing synaptic noise. When glutamate is released from vesicles into the synapse, ELFN prevents hyperactivation of mGluRs, which could prevent propagation of action potentials. Excessive glutamatergic signaling triggers cooperativity, which may help recruit more mGluR-ELFN complexes to synapses to further strengthen the autoinhibitory loop, reigning in glutamate release further.

Crucially, this model helps explain disease mechanisms where this regulation is disrupted. Our analysis of mGluR mutations indicated at least four orthogonal mechanisms shared between diseases can occur. First, mutations can simply disrupt the stability of mGluRs or ELFNs to the point that they cannot express (mGluR7 K689fs). Second, mutations can destabilize mGluRs so they are not properly processed and trafficked to the membrane (mGluR6 G150S, I405T, mGluR7 I154T, R197C, W310C, R658W, and T675K). Third, the mutations can also alter mGluR responsiveness to glutamate (mGluR7 R197C, R622Q, R658Q, E891K, A85S, I154T, E394K, and G471A). Last, mutations can interfere with binding and recruitment of mGluR by ELFN (mGluR6 R352C, mGluR7 E394K, and ELFN1 P50H). This would prevent mGluR from properly localizing to synapses, an indicator of severe neurological phenotypes (*3*, *19*). There is a final possibility suggested by this work, wherein ELFN and mGluR still bind each other and mGluR localizes to the synapse, but ELFN allosteric regulation of mGluR is altered. Additional work is needed to characterize conditions leading to such a scenario, but such a case could hold therapeutic potential. Once mGluR and ELFN are properly localized at mature synapses, their allosteric interactions could be dynamically and temporarily adjusted by therapeutics to control group III mGluR activity and rescue disease states.

## MATERIALS AND METHODS

### Bacmid generation, expression, and purification of mGluR7

A fusion construct of human mGluR7 (residues 1 to 859) and enhanced green fluorescent protein, Flag, and His10 tags connected by an HRV-3C protease site were inserted into modified pEG BacMam vector (*69*). The BacMam was then transfected into Sf9 cells (Thermo Fisher Scientific, 11496015) to produce baculovirus. The supernatant of the third generation of virus production (P3) was then harvested and concentrated 10× in FreeStyle 293 expression medium (Thermo Fisher Scientific, 12338018).

HEK293S GnTi^−^ cells [800 ml; American Type Culture Collection (ATCC), CRL-3022] were grown at 37°C with 8% CO_2_ to a confluency of 2.0 × 10^6^ to 3.0 × 10^6^ cells/ml in FreeStyle 293 expression medium supplemented with 2% fetal bovine serum (FBS) and infected with 12 ml of 10× P3 baculovirus. After 18 hours, sodium butyrate (Sigma-Aldrich, B5887) was added to 10 mM. The cells were then incubated for a further 48 hours and pelleted by centrifugation and flash frozen.

The mGluR7 homodimers were then isolated in detergent-free self-extracting nanodiscs (*25*). Hex-20B1WA peptide (synthesized by GenScript) was first dissolved in *N*,*N*′-dimethylformamide (100 mg/ml) and then diluted to 1 mg/ml in phosphate-buffered saline (PBS) and supplemented with 0.5 μM phenylmethylsulfonyl fluoride, 1× Halt Protease Inhibitor Cocktail, EDTA-free, and 50 μM MMPIP (Sigma-Aldrich, M3074). The cell pellets were thawed and resuspended in 50 ml of this lysis buffer. After the cells were disrupted by sonication, they were incubated at 4°C overnight with gentle agitation. Insoluble matter was removed by centrifugation, and the clarified supernatant was incubated with Pierce M2 anti-Flag affinity resin (Millipore, A2220) for 1 hour at 4°C with gentle agitation. The beads were washed with 50 mM tris-HCl (pH 8.0), 400 mM NaCl, and 5% glycerol and eluted with 3xFlag peptide (0.25 mg/ml; GenScript) in wash buffer. The eluted protein was then concentrated to 1.5 mg/ml in an Amicon Ultra Centrifuge Filter (MWCO, 100 kDa). All samples underwent further purification on a Superdex 75 Increase 10/300 GL (GE Healthcare) equilibrated in 50 mM tris-HCl (pH 8.0), 400 mM NaCl, and 5% glycerol to obtain homogeneous samples. Peak fractions containing the protein of interest were pooled, concentrated to 1 to 2 mg/ml, flash frozen in liquid nitrogen, and stored at −80°C until usage.

### Bacmid generation, expression, and purification of ecto-ELFN2

A fusion construct of human codon-optimized human ELFN2 (residues 1 to 379; UniProt, Q5R3F8) and Fc [immunoglobulin G1 (IgG1) (heavy chain) residues 233 to 462, OP142478], Flag, and His10 tags connected by an HRV-3C protease site were inserted into modified pEG BacMam vector by a commercial gene synthesis company (GenScript). The BacMam was then transfected into Sf9 cells to produce baculovirus as above.

HEK293S GnTi^−^ cells (1.6 liters) were grown to a confluency of 2.0 × 10^6^ to 3.0 × 10^6^ cells/ml in FreeStyle 293 expression medium supplemented with 2% FBS and infected with 12 ml of 10× P3 baculovirus. After 18 hours, sodium butyrate was added to 10 mM. The cells were then incubated for a further 120 hours and pelleted by centrifugation.

The supernatant was then passed through a 0.22-μm filter and mixed with 1 ml of Pierce anti-DYKDDDDK affinity resin (Thermo Fisher Scientific, A36801) for 1 hour at 4°C with vigorous shaking. The resin was then collected and washed with 10 ml of 10 mM tris-HCl (pH 8.0) and 500 mM NaCl (buffer A). The resin was further washed with buffer A and 10% glycerol, followed by 2 ml of buffer A without glycerol. The protein was then eluted from the resin with 6 ml of buffer A with 3xFlag peptide (0.33 mg/ml; GenScript, RP21087). The elution was then concentrated to 3.5 mg/ml on an Amicon Ultra-4 centrifugal filter (Sigma-Aldrich, UFC805024) and buffer exchanged with buffer A. The sample was analyzed by SDS–polyacrylamide gel electrophoresis (PAGE), aliquoted, and flash frozen.

### Cryo-EM sample preparation, image acquisition, and processing

For complex formation, mGluR7 was incubated at a molar ratio of 2:1 with ecto-ELFN2, 50 μM MMPIP, and PreScission Protease for 30 min at 4°C per the manufacturer’s instructions. The complex was centrifuged before grid preparation. Three microliters of mGluR7-ELFN2 complex was applied to freshly glow-discharged 300-mesh copper grids (CF-1.2/1.3) inside a FEI Vitrobot Mark IV (Thermo Fisher Scientific) that was set at 5°C and 100% humidity. A blot force of 0, blot time of 3 s, and wait time of 6 s were used before blotting and plunge freezing in liquid ethane. Cryo-EM images of the mGluR7-ELFN2 complex were acquired on a JEOL CryoARM300 equipped with a Gatan K3 direct electron detection camera in counting mode at a magnification of ×50,000, giving a nominal pixel size of 0.7753 Å. A total of 18,728 movies were collected with a defocus range of −1.0 to −2.0 μm. A total dose of 50 e^−^ Å^−2^ was attained across 50 frames for a 2.5-s total exposure time (*70*, *71*).

### Image processing and 3D reconstruction

The dataset was processed with CryoSPARC (fig. S1A) (*72*). Movies were motion corrected for beam-induced motion, and the contrast transfer function (CTF) estimation was performed with the patch CTF estimation tool on motion-corrected images in CryoSPARC. We used the CryoSPARC TOPAZ program for particle selection and extraction (*73*), which resulted in a total of 239,195 particles. Selected particles were subjected to ab initio reconstruction, which was followed by heterogeneous and homogeneous and nonuniform (NU) refinement. A set of 96,225 particles was reextracted with local motion correction to further improve the density at the extracellular regions and was further subjected to ab initio reconstruction. After heterogeneous refinement, a set of 28,023 particles was chosen and subjected to NU refinement, resulting in a final resolution of ∼5.0 Å. We then performed local refinements with a mask on the ECD domains, resulting in a resolution of 4.6 Å for the mGluR7 VFT and ELFN2 LRR domains (*70*, *71*). The reported resolutions of the final maps were estimated with the gold-standard Fourier shell correlation (FSC) in CryoSPARC (*72*). The local resolution predictions were calculated by local resolution estimation with CryoSPARC, with the resolution range estimated with the FSC of 0.143 criterion (*74*). EM density visualization was performed in ChimeraX (*75*).

### Model building and refinement

We used the inactive structure of mGluR7 homodimer (*23*) and the ELFN2 LRR domain models predicted by AlphaFold to initially dock into the cryo-EM map with UCSF Chimera (*76*). Two protomers of each model were fitted onto the cryo-EM density and then were manually adjusted, built, and real-space refined in Coot (*77*). The local resolution in the CRD and TMD was relatively low, so the model could not be built confidently. The locally refined mGluR7-ELFN2 model was subjected to iterative manual building in Coot, which was followed by real-space refinement in PHENIX against the corresponding sharpened map using global minimization, local rotamer fitting, and restrained group atomic displacement parameter refinement. The resulting model was subsequently validated with comprehensive validation (cryo-EM) in PHENIX (*78*). Structures were visualized, and figures were prepared in UCSF Chimera (*76*) and ChimeraX (*75*). The data collection and refinement statistics are listed in table S1.

### Molecular visualization

Models of ELFN1, mGluR4, mGluR6, and mGluR8 were generated from the mGluR7-ELFN2 model by mutating residues near the mGluR7-ELFN2 interface using the rotamer tool in ChimeraX (*75*).

### cDNA constructs

WT mGluR constructs were obtained from the Missouri S&T cDNA Resource Center (GRM2, catalog no. GRM2000000; GRM4, catalog no. GRM4000000; GRM6, catalog no. GRM6000000; GRM7, catalog no. GRM7000001; and GRM8, catalog no. GRM8000000). All mGluR and ELFN mutants were generated using conventional site-directed mutagenesis. Ecto-ELFN1-Fc and Ecto-ELFN2-Fc constructs were synthesized using the ectodomain residues with human codon optimization and a C-terminal Fc fusion and were inserted into the mammalian expression vector pEG. Venus-156–239-Gβ1, Venus-1–155-Gγ2, and masGRK3ct-Nluc-HA constructs were described previously (*79*). All constructs were confirmed by Eurofins Nanopore sequencing.

### Cell culture

HEK293T/17 cells (ATCC, CRL-11268) were cultured in Dulbecco’s modified Eagle’s medium (DMEM) supplemented with 10% FBS, minimum essential medium nonessential amino acids (Life Technologies), and 1 mM sodium pyruvate, at 37°C in a humidified incubator containing 5% CO_2_. For each experiment, cells were seeded in 3.5-cm dishes at a density of 1 × 10^6^ cells per dish and transfected 4 to 6 hours later. Transient transfections were performed using PEI or Metafectene Pro (Biontex Laboratories GmbH), according to the manufacturer’s instructions.

### Protein G pull-down

Fc tag recombinant proteins were produced in HEK293T/17cells. The day after transfection, the culture medium was replaced with OPTI-MEM and incubated at 30°C to promote accumulation of secreted proteins. After 48 hours, medium was collected and incubated lysates from mGluR-transfected cells in 1% Triton X-100 lysis buffer, rotating for ∼1 hour at 4°C. Protein G Sepharose Beads (Cytiva) were then added and incubated for an additional ∼1 hour at 4°C. Samples were washed three times with 1% Triton X-100 lysis buffer. Proteins were eluted with SDS sample buffer at 42°C for 10 min. SDS-PAGE and Western blotting were performed using 5% milk blocking and then anti-mGlu4 (Invitrogen, PA550999), anti-mGlu6 (Thermo Fisher Scientific, 50-254-4596), anti-mGlu7 antibody (Invitrogen, PIMA531754), anti-mGlu8 (Invitrogen, PA5-49742), or anti-Fc antibody (Invitrogen, MA1-10378) in 5% milk at 1:1000. Secondary antibodies were purchased from Jackson ImmunoResearch and used at 1:2000 in 5% milk. Images were obtained using a KwikQuant Imager and KwikQuant ECLSolutions (Kindle Biosciences).

### Real-time kinetic BRET assays for G protein activation

Cells were transfected in 3.5-cm dishes with 0.25 μg each of GαoA, Venus 156–239-Gβ1, Venus 1–155-Gγ2, and masGRK3ct-Nluc-HA, using Metafectene Pro. At the time of transfection, 0.1% Matrigel (Corning, catalog no. CB-40230A) was added to the culture medium. Two days posttransfection, cells were harvested, washed three times with PBS, and seeded into 96-well white flat-bottom microplates (Greiner Bio-one) in Tyrode’s solution (Thermo Fisher Scientific, catalog no. AAJ67593AP) at a density of 5 × 10^4^ to 10 × 10^4^ cells per well. The NanoLuc substrate (Nano-Glo Luciferase Assay Reagent; Promega) was diluted 1:250 in Tyrode’s solution and added to the wells.

For transcellular mGlu-ELFN signaling assay, cells were transfected separately into two groups: (i) cells expressing mGluR and biosensor constructs; and (ii) cells expressing either an empty pcDNA3.1 vector (control), ELFN1, or ELFN2 without any biosensor. Two days after expression, mGluR- and biosensor-expressing cells were mixed with empty vector- or ELFN-expressing cells at a 1:2 ratio and coincubated for 2 hours.

Glutamate was applied at 300 μM for mGlu2, mGlu4, or 10 mM for mGlu7, in Tyrode’s solution, with BRET signal acquisition every 100 ms using a PHERAstar FSX microplate reader (BMG Labtech). BRET was calculated as the ratio of light emitted at 535 nm (Venus-Gβ1γ2) to that at 475 nm (masGRK3ct-Nluc-HA). G protein activation was determined as the change in BRET ratio upon Glutamate addition. All measurements were performed at 37°C.

### Fluorescence-activated cell sorting ELFN-binding assay

Cells were transfected with mGluR constructs (2 μg per well) in 3.5-cm dishes with Metafectene Pro (3 μl per well). Two days after transfection, cells were collected and blocked with 0.1% bovine serum albumin–PBS for 1 hour at 4°C. Bv421–antihuman IgG Fc antibody (BioLegend, catalog no. 410704) and various concentrations of Ecto-ELFN1/2-Fc were preincubated for 1 hour at 4°C and then added to 1 × 10^6^ mGluR-expressing cells in a total volume of 300 μl, rotating for an additional 1 to 2 hours at 4°C in the dark. After two washes, cells were analyzed using a BD Symphony flow cytometer, and data were processed with FlowJo software.

For ligand effects on ELFN-mGluR interactions, 0.3 μM Ecto-ELFN1-Fc and 0.03 μM Ecto-ELFN2-Fc were used for mGluR4; 0.1 μM Ecto-ELFN1-Fc and 0.01 μM Ecto-ELFN2-Fc were used for mGluR6, mGluR7, and mGluR8. All ligands were applied at saturating concentrations: 1 mM glutamate for mGluR4, mGluR6, and mGluR8 or 10 mM for mGluR7; 1 mM L-AP4; 100 μM CPPG; 10 μM ADX88178; 10 μM ADX71743; 100 μM PHCCC; 100 μM VU0364770; 10 μM Valiglurax; 10 μM LuAf21934; 100 μM VU0080241; 10 μM VU0155041; 10 μM MPEP; 10 μM VU0422288; 10 μM AMN082; 10 μM MMPIP; and 100 μM XAP044.

### Synaptosomal pull-down assay

Mouse brains were homogenized in 0.32 M sucrose, 5 mM Hepes, and protease inhibitor (1×) using a Potter homogenizer (4 ml of buffer per brain). Samples were centrifuged at 1000*g* for 10 min at 4°C, and the supernatant (S1) was collected. The pellet (P1) was rehomogenized in the same buffer volume and centrifuged again at 1000*g*, and the resulting supernatant (S1′) was combined with S1. The pooled supernatants were centrifuged at 12,000*g* for 20 min to obtain pellet P2. P2 was resuspended in 4 ml of homogenization buffer and centrifuged again at 12,000*g* for 20 min, yielding pellet P2′. P2′ was resuspended in 750 μl of homogenization buffer and loaded onto a sucrose step gradient (0.85/1.0/1.2 M, 1.3 ml each layer). Gradients were centrifuged at 85,000*g* for 2 hours at 4°C, and synaptosomes were collected from the 1.0/1.2 M interface. Synaptosomes were dissolved in IP buffer (2% LMNG, 300 mM NaCl, 50 mM Hepes, and 1× protease inhibitor).

Synaptosomes from one mouse brain were divided into four conditions: Fc, Fc and L-AP4, Ecto-ELFN2-Fc, and Ecto-ELFN2-Fc and L-AP4. Protein G Sepharose beads (Cytiva) were preincubated with Ecto-ELFN2-Fc or Fc proteins for 1 hour at 4°C and then added to synaptosome extracts for an additional 1 to 2 hours. Final concentrations were 0.1 μM for Ecto-ELFN2-Fc/Fc and 1 mM for L-AP4. Samples were washed three times with IP buffer, and proteins were eluted with SDS sample buffer at 42°C for 10 min.

### Cell surface biotinylation

mGluR7 mutant surface proteins were biotinylated using the Pierce Cell Surface Protein Biotinylation and Isolation Kit (Thermo Fisher Scientific, A44390) according to the manufacturer’s instructions. Approximately 2 × 10^7^ cells were used for each condition. Cells were washed once with 15 ml of PBS and then incubated with 10 ml of Sulfo-NHS-SS-Biotin (0.25 mg/ml) for 10 min at room temperature. Cells were washed twice with 15 ml of ice-cold TBS and lysed in lysis buffer on ice for 30 min. Lysates were cleared by centrifugation at 15,000*g* for 5 min, and a small fraction was saved for total protein analysis.

The remaining lysates were incubated with NeutrAvidin agarose for 30 min at room temperature, washed four times, and eluted with 200 μl of 10 mM dithiothreitol elution buffer for 30 min at room temperature. Eluates were collected by centrifugation, and both eluates and total lysates were mixed with β-mercaptoethanol–containing sample buffer and heated at 42°C for 10 min. SDS-PAGE and Western blotting were performed, and band intensities were quantified using Fiji software.

### Cell culture and transfection for smFRET experiments

Expression vector for mGluR4 with a C-terminal Flag-tag in pcDNA3.1^+^ vector was purchased from GenScript. Mutation of amino acid E568 to the amber codon (TAG) for unnatural amino acid incorporation was performed using the QuikChange site-directed mutagenesis kit (QIAGEN). The plasmid was verified by full-length sequencing (Plasmidsaurus).

HEK293T cells (Sigma-Aldrich, 41106514) were maintained in growth medium DMEM (Corning), 15 mM Hepes (pH 7.4, Gibco), 10% (v/v) FBS (GE Healthcare), and penicillin-streptomycin (100 U/ml; Gibco). Cells were kept at 37°C under 5% CO_2_ and passaged with 0.05% trypsin-EDTA (Gibco) when they reached 80% confluency.

HEK293T cells were seeded on poly-d-lysine–coated coverslips in 12-well plate 24 hours before transfection. Two hours before transfection, the growth medium was changed to fresh growth medium containing 0.25 mM unnatural amino acid trans-cyclooct-2-en–l-lysine (SiChem). Cells were cotransfected with mGluR4 plasmid and tRNA-containing plasmid pNEU-hMbPylRS-4xU6M15 (gift from I. Coin; Addgene, plasmid no. 105830) at 1:1 w/w (total 2 μg of plasmid per well). Lipofectamine 3000 (Thermo Fisher Scientific) was used as transfection agent. After 24 hours, the medium was exchanged, supplemented with 0.25 mM unnatural amino acid. Last, after total 48-hour expression, the medium containing unnatural amino acid was removed, cells were washed twice with extracellular buffer [128 mM NaCl, 2 mM KCl, 2.5 mM CaCl_2_, 1.2 mM MgCl_2_, 10 mM sucrose, and 10 mM Hepes (pH 7.4)] and incubated for 30 min in growth medium (without unnatural amino acid). The medium was then removed, and cells were washed one more time with extracellular buffer before addition of Cy3 and Cy5 tetrazine dyes (Click Chemistry Tools) to the final concentration of 25 μM each in the extracellular buffer. After 10 min in the labeling solution, glutamate was added to the cells at final concentration of 500 μM followed by another 5-min incubation. Last, the cells were washed in extracellular buffer to remove the excess dye and incubated in DPBS, followed by gentle pipetting to detach the cells. Cells were then pelleted by centrifugation at 5800*g*, at 4°C for 5 min. Cells were then resuspended in 120 μl of lysis buffer (150 mM NaCl, 50 mM tris, and protease inhibitor cocktail) and 0.1% LMNG-CHS (10:1) with 0.1% GDN (pH 7.35). The sample was left on gentle mixing for 1 hour at 4°C and then centrifuged at 20,000*g* at 4°C for 20 min. The supernatant was collected and kept on ice until imaging.

### Single-molecule FRET imaging

Single-molecule FRET experiments were performed in glass flow chambers constructed from glass coverslips (VWR) and slides (Thermo Fisher Scientific) passivated with mPEG (Laysan Bio) and 1% (w/w) biotin-PEG. Right before experiments, the glass surface was functionalized by 2-min incubation with 500 nM NeutrAvidin (Thermo Fisher Scientific) and then washed with T50 buffer [50 mM NaCl, 10 mM tris (pH 7.4)], followed by addition of 20 nM biotinylated anti-FLAG antibody (A01429, GenScript) for 30 min. Excess antibody was removed by washing with T50 buffer.

Protein sample was then added to the flow chamber, and, after achieving adequate particle density, the flow chamber was thoroughly washed with wash buffer [50 mM NaCl, 10 mM tris, and 0.005% LMNG-CHS (10:1) with GDN (0.005%, w/v) (pH 7.4)]. Last, imaging buffer consisting of 4 mM Trolox, 128 mM NaCl, 2 mM KCl, 2.5 mM CaCl_2_, 1.2 mM MgCl_2_, 40 mM Hepes, 0.005% LMNG-CHS (10:1) with GDN (0.005%, w/v), 4 mM protocatechuic acid (Sigma-Aldrich), and bacterial protocatechuate 3,4-dioxygenase (1.6 U/ml) (Oriental Yeast), and pH 7.35 was added to the chamber to perform the fluorescence imaging. All buffers and reagents dilutions were prepared using ultrapure water (Invitrogen). CPPG (Tocris) was dissolved in 100 mM NaOH at 100 mM stock concentration. For the apo state, CPPG was added to the imaging buffer at a final concentration of 1 mM to block any possible confounding effect from trace glutamate potentially present in the buffer.

Data were collected on a homebuilt total internal reflection fluorescence microscope with 100× objective (Olympus, 1.49 numerical aperture, oil-immersion) using an excitation filter set with a quad-edge dichroic mirror (Di03-R405/488/532/635, Semrock) and a long-pass filter (ET542lp, Chroma), and, in emission pathway, a dichroic beam splitter (FF640-FDi01, Semrock) was used to split the donor and acceptor signals. Donor (Cy3) was excited with a 532-nm laser, and emission signals from both Cy3 and Cy5 fluorophores were simultaneously recorded with an electron multiplying charge-coupled device (iXon Ultra, Andor) with 50-ms time resolution. Lasers at wavelength of 532 and 638 nm (RPMC Lasers) were used for donor and acceptor excitation, respectively.

### smFRET data analysis

Single-molecule fluorescence data were analyzed using smCamera (*80*), custom MATLAB (MathWorks) scripts. Statistical analysis was done in OriginPro (OriginLab). Particle detection and generation of raw FRET traces were done automatically using the smCamera software. Only particles with present acceptor signal upon donor excitation and acceptor brightness greater than 10% above background with Gaussian intensity profile were selected. From this initial selection, particles that exhibited a single-donor and single-acceptor bleaching step during the acquisition time, that presented anticorrelated donor and acceptor intensity behavior and stable total intensity (ID + IA), and that lasted for more than 5 s were manually selected for further analysis (∼20 to 30% of total molecules per movie). Apparent FRET efficiency was calculated as (IA − 0.088ID)/(ID + IA), where ID and IA are raw donor and acceptor intensities, respectively. Population smFRET histograms were generated by compiling at least 200 FRET traces from each experiment. FRET state occupancy was obtained using Gaussian multipeak fitting on population smFRET histograms in OriginPro. State occupancy is measured as the area of specified peaks relative to the total area. FRET traces were idealized using a hidden Markov model implemented in vbFRET software. Idealized traces were used to generate the dwell-time distribution histograms. Dwell times were calculated by fitting a single exponential decay function to the dwell-time distributions. The high-FRET state was defined as FRET > 0.78, the low-FRET state was defined as FRET < 0.22, and transitions were defined as ΔFRET > 0.10. Average dwell times as a function of glutamate, CPPG, and/or ecto-ELFN2 were calculated using a single exponential fit to dwell-time histogram.

### Coimmunoprecipitation of full-length ELFN1-myc mutant

For each experiment, four populations of HEK293 cells were transiently transfected with 15 μg of the following: (i) mGluR-expressing cells, (ii) pcDNA3.1-expressing (control) cells, (iii) ELFN1-myc-expressing cells, and (iv) ELFN1-P50H-myc–expressing cells using PEI transfection reagent. Cells were lysed using 1% Triton X-100 lysis buffer and combined. After ∼1 hour of rotation at 4°C, lysates were incubated with Protein G Beads conjugated to c-Myc antibody (GenScript, catalog no. A00172) for ∼1 hour at 4°C and washed three times with centrifugation and fresh lysis buffer. Proteins were eluted using β-mercaptoethanol–containing sample buffer, and SDS-PAGE was performed followed by Western blotting for inputs and immunoprecipitated proteins.

The following antibodies were used for Western blotting and their dilutions used in 3% milk: c-Myc, 1:1000 (GenScript, catalog no. A00704); HA (mGluR8), 1:1000 (GenScript, catalog no. A01963); mGluR7, 1:1000 (EMD Millipore catalog no. 07–239); and mGluR4, 1:1000 (Invitrogen, catalog no. MA5-27293). Secondary antibodies were purchased from Jackson ImmunoResearch and used at 1:10,000 in 3% milk. mGluR and ELFN1 constructs were described previously (*5*, *17*).

### Bioinformatics

Multiple-sequence alignment was performed between human proteins (UniProt entry): ELFN1 (P0C7U0) and ELFN2 (Q5R3F8), as well as between mGluR2 (Q14416), mGluR4 (Q14833), mGluR5 (P41594), mGluR6 (O15303), mGluR7 (Q14831), and mGluR8 (O00222) using the CLUSTAL Omega (1.2.4) algorithm.

### MD simulations

The cryo-EM structure obtained in this study was used as the starting model for all MD simulations. The mGluR7 dimer was extracted from the mGluR7-ELFN2 cryo-EM structure and used as the starting structure for apo simulations, enabling direct comparison of mGluR7 conformational dynamics in the presence and absence of ELFN2. All simulations were carried out using GROMACS 2025.2 (*81*), in combination with the CHARMM36-jul2022 force field and the TIP3P water model (*82*).

The apo-mGluR7 (containing residues 38 to 521) system was solvated in a cubic simulation box with an approximate side length of 13.29 nm, corresponding to a total volume of ∼2346 nm^3^. A buffer distance of 1.0 nm was maintained between the solute and the box boundaries. To ensure charge neutrality and mimic physiological conditions (150 mM KCl), 230 K^+^ and 212 Cl^−^ ions were introduced. All amino acid residues and terminal groups were assigned standard protonation states corresponding to pH 7.

The mGluR7_38-521_–ELFN2_23-248_ system was constructed in a cubic box with side lengths of ∼16 nm, corresponding to a volume of ∼4114 nm^3^. The system was neutralized by addition of 398 K^+^ and 372 Cl^−^ ions and adjusted to a final ionic strength of 150 mM KCl.

Energy minimization was conducted using the steepest descent algorithm (*81*) until the maximum force reached below 1000 kJ/mol·nm. Subsequently, the systems were equilibrated for 100 ps under NVT conditions at 300 K, followed by 100 ps under NPT conditions at 1 bar. During equilibration, positional restraints on heavy atoms were applied. Temperature coupling was maintained at 300 K using the V-rescale thermostat (*83*), while pressure was controlled using the C-rescale barostat with isotropic coupling (*84*).

All bonds involving hydrogen atoms were constrained using the LINCS algorithm (*85*), enabling a 2-fs integration time step. Long-range electrostatic interactions were computed using the particle mesh Ewald method (*86*). Short-range nonbonded interactions were evaluated with a 1.2-nm cutoff for both Coulomb and van der Waals terms. A force-switching scheme was applied for van der Waals interactions between 1.0 and 1.2 nm. Neighbor searching was performed using the Verlet scheme, and periodic boundary conditions were applied in all spatial directions (*87*).

All simulations were analyzed using GROMACS 2025.2 (*81*). RMSD and root-mean-square fluctuation analyses were performed on mGluR7 backbone atoms for each replicate of both apo and ELFN2-bound systems to assess structural stability and flexibility. Equilibration was evaluated from RMSD trajectories, and the first 170 ns (apo) and 190 ns (ELFN2 bound) were excluded on the basis of lack of major structural change.

Clustering analysis was performed on mGluR7 using the GROMOS algorithm (*88*) via gmx cluster in GROMACS with a backbone RMSD cutoff of 0.35 nm over the last 500 ns of each simulation. Clustering was performed separately for each replicate and for concatenated apo and mGluR7-ELFN2 trajectories. Cluster centroids were used to extract representative structures and characterize dominant conformational states.

Residue-residue distance maps at the mGluR7-ELFN2 interface were computed using the GROMACS tool gmx mdmat on the initial structure and on the last 500 ns of each replicate trajectory. Contact frequencies were then calculated using custom Python scripts using the MDAnalysis package (*89*) and saved as NumPy binary files. The resulting contact maps were visualized using in-house Python plotting scripts.

### Statistical analysis

All statistical analyses were performed using GraphPad Prism version 10.4. Data are presented as means ± SEM. Each point represents three technical replicates unless otherwise stated. Comparisons were made using two-tailed, Student’s *t* tests unless specified otherwise in the figure legends when comparing the means of two independent groups. Two-way analysis of variance (ANOVA) with Tukey’s multiple comparisons test was used to analyze data with two categorical independent variables and one continuous dependent variable when comparing all possible pairs of group means. Significant difference was expressed as *P < 0.05, **P < 0.01, ***P < 0.001, and ****P < 0.0001. Not significant (n.s.) was indicated where appropriate.
