# Supplementary material for: Structural basis for modulation of group III mGlu receptors by transsynaptic interactions
Source: Sci Adv. 2026 Sep 4;12(36):eaed3793. doi: 10.1126/sciadv.aed3793 (PMC13544217; doi:10.1126/sciadv.aed3793)
Supplement: Supplementary file 1 [file sciadv.aed3793_sm.pdf]

Science Advances
AAAS

Supplementary Materials for

Structural basis for modulation of group III mGlu receptors by transsynaptic interactions

William G. Ludlam *et al.*

Corresponding author: Kirill A. Martemyanov, kmartemyanov@miami.edu

Sci. Adv. **12**, eaed3793 (2026)
DOI: 10.1126/sciadv.aed3793

The PDF file includes:

Figs. S1 to S15
Table S1
Legend for movie S1

Other Supplementary Material for this manuscript includes the following:

Movie S1

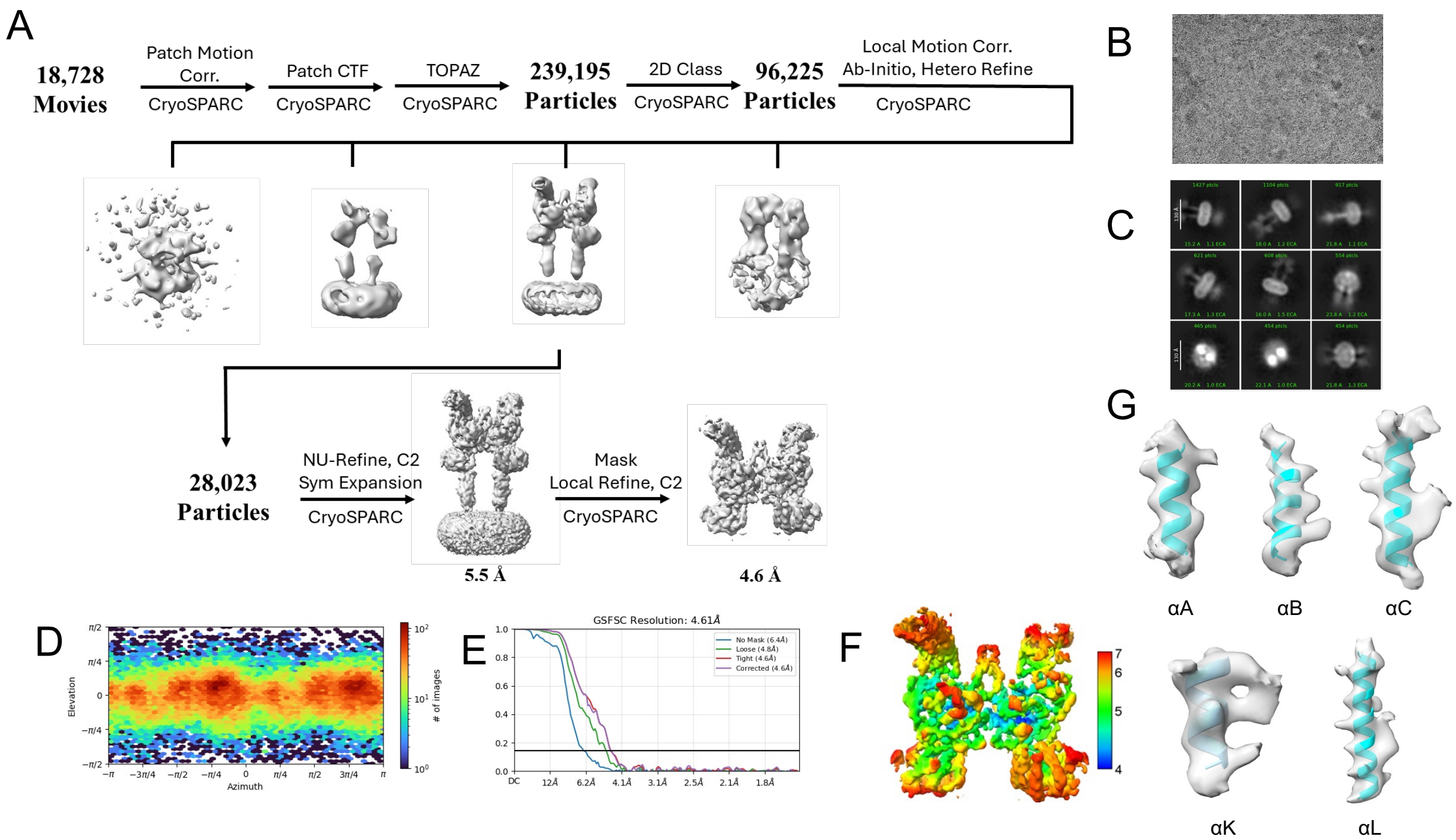

Supplementary Figure 1. Cryo-EM processing for ecto-ELFN2-mGluR7.
A) Flowchart showing steps in cryo-EM data processing. B) Representative cryo-EM micrograph. C) Representative 2D class averages. D) Euler angle orientation distribution plots. E) Gold Standard Fourier shell correlation (FSC) curves. F) Global density maps colored according to local resolution. G) Representative α-helices.

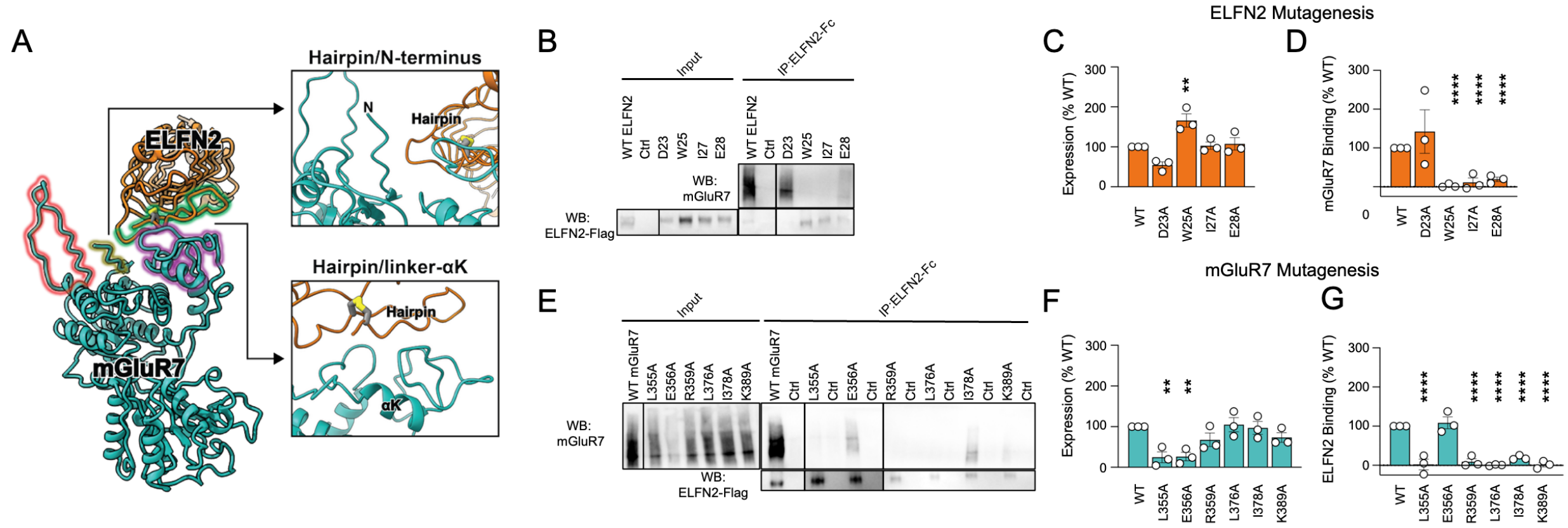

Supplementary Figure 2. Key regions involved mGluR7-ELFN2 interface.

A) Regions include ELFN2 hairpin/N-terminus (green highlight), mGluR7 helix αK and αK–αL loop (purple highlight), mGluR7 N-terminus (goldenrod highlight), and mGluR7 T137–V143 homodimer interface loop (salmon highlight). B) Representative immunoblots of ecto-ELFN2 alanine mutations input (right) and mGluR7 co-immunoprecipitation (left). C) Quantifications of band intensities for input and D) co-immunoprecipitation, normalized for input. E) Representative immunoblots of mGluR7 alanine mutations input (right) and ecto-ELFN2 co-immunoprecipitation (left). F) Quantifications of band intensities for input and G) co-immunoprecipitation, normalized for input. Data are ± SEM of three individual experiments. *$p<0.05$; **$p<0.01$; ***$p<0.001$; ****$p<0.0001$ (Ordinary one-way ANOVA with Dunnett's multiple comparisons test).

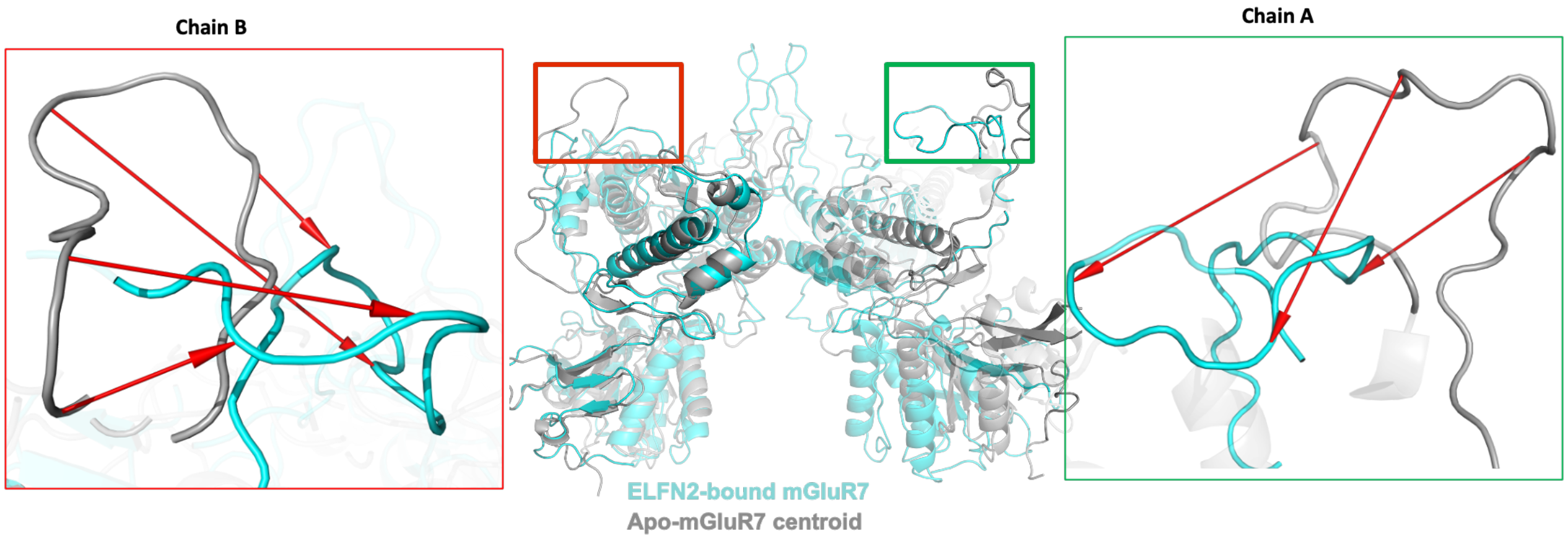

Supplementary Figure 3. Structural comparison of apo and ELFN2-bound mGluR7 reveals displacement of the extracellular ELFN2-binding loop at the receptor–ELFN2 interface. The ELFN2-bound cryo-EM structure of mGluR7 (cyan) is aligned with the centroid of the most populated cluster (Cluster 1) from the apo ensemble (gray). The apo centroid was obtained from a concatenated trajectory of three independent MD simulations. Close-up views of mGluR7 chains A and B highlight the ELFN-binding loop (residues L372–G392). Arrows indicate ELFN2-binding loop displacement consistent with an open conformation in the Apo state and a closed conformation in the ELFN2-bound complex.

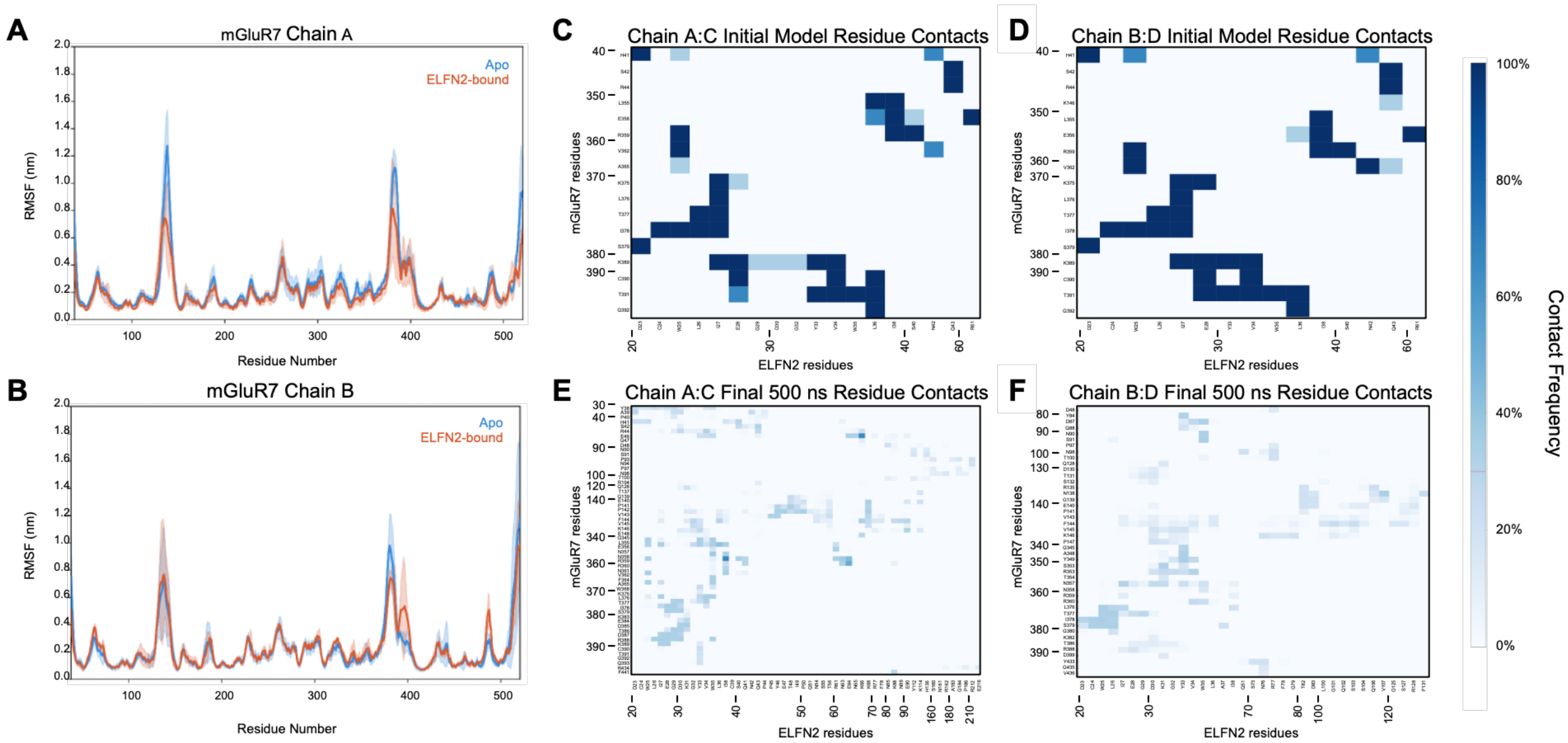

Supplementary Figure 4. ELFN2 binding increases stability of mGluR7 at binding interface. A) Residue-level flexibility of mGluR7 chain A and B) chain B in the absence and presence of ELFN2. Mean RMSF ± standard deviation across three independent simulations for apo and ELFN2-bound mGluR7 chain A. C) Protomer A:C interface at the start of simulation and D) averaged over the final 500 ns. E) protomer B:D interface at the start of simulation and F) averaged over the final 500 ns.

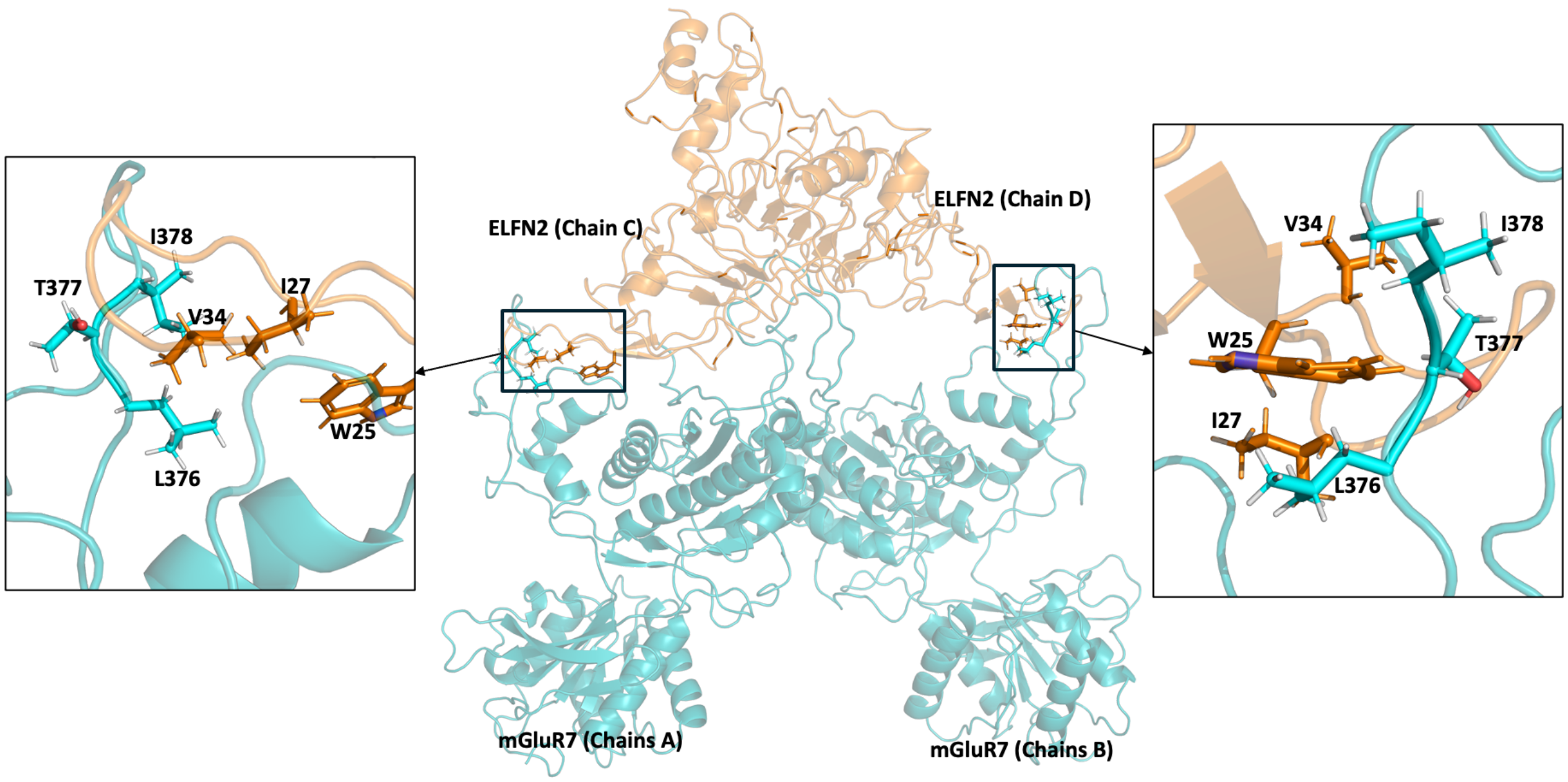

Supplementary Figure 5. Key residues involved in the mGluR7–ELFN2 interface. The centroid structure of the most populated cluster (Cluster 1) was obtained from a concatenated trajectory of three independent MD simulations of the mGluR7–ELFN2 complex. The core hydrophobic interactions at the mGluR7–ELFN2 interface are conserved.

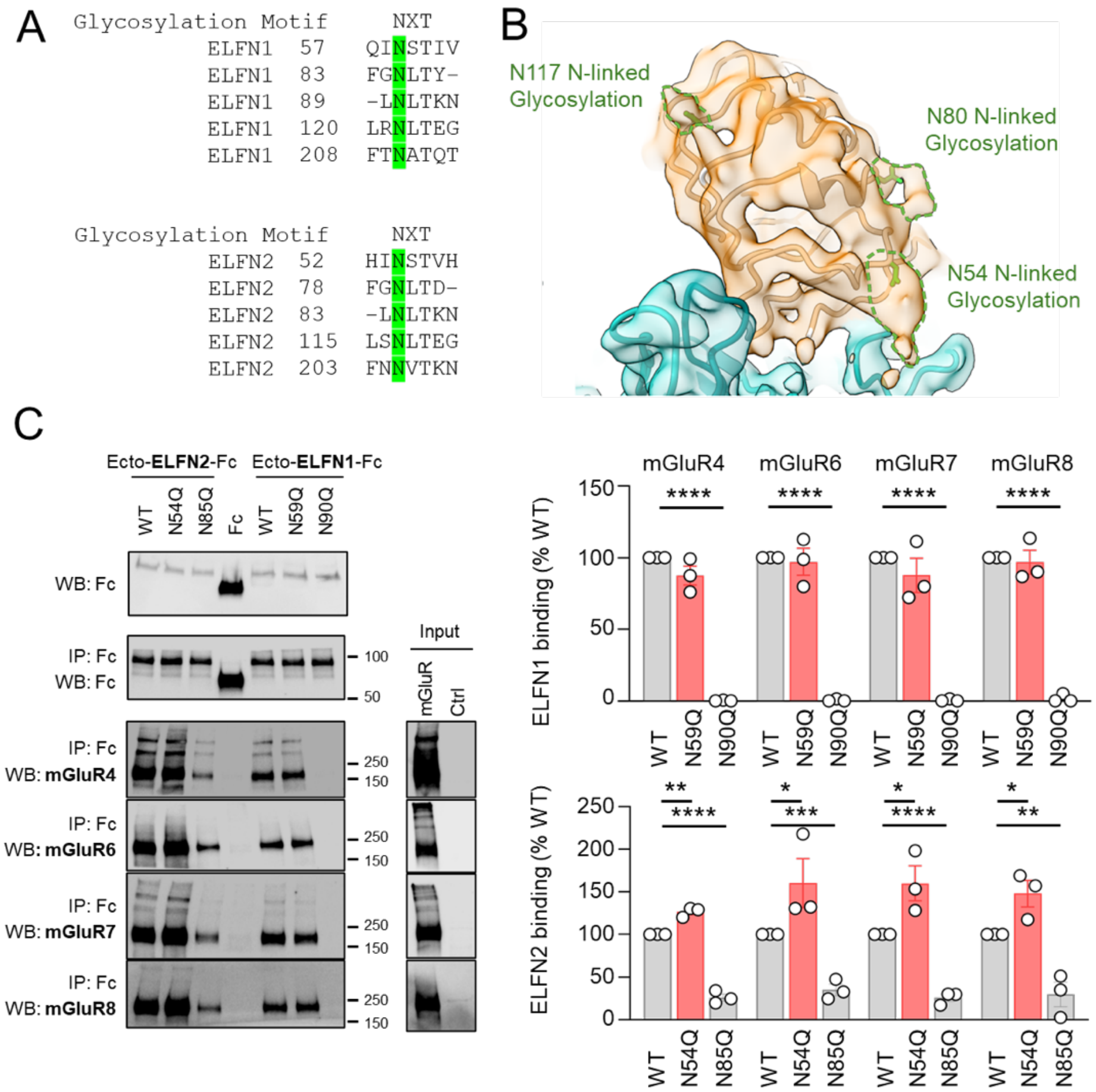

Supplementary Figure 6. N-linked glycosylation sites on ELFN contribute to mGluR binding interface.

A) Protein sequence alignment for ELFN1 and 2 at each of the five NXT consensus motifs marking potential glycosylation sites. B) Additional electron density probability is present near N54, N80 and N117 on ELFN2. Note: the electron-density surface threshold is not the same as in Fig. 2C. C) Representative immunoblots of mGluR input (right), glycosylation mutant ecto-ELFN input (top) immunoprecipitation of ecto-ELFN2-Fc (second from top), and co-immunoprecipitation of group III mGluR. Quantifications of band intensities. Part of these data are shown in Fig. 2F. Data are means ± SEM of three individual experiments. *p<0.05; **p<0.01; ***p<0.001; ****p<0.0001 (unpaired Student's t-test).

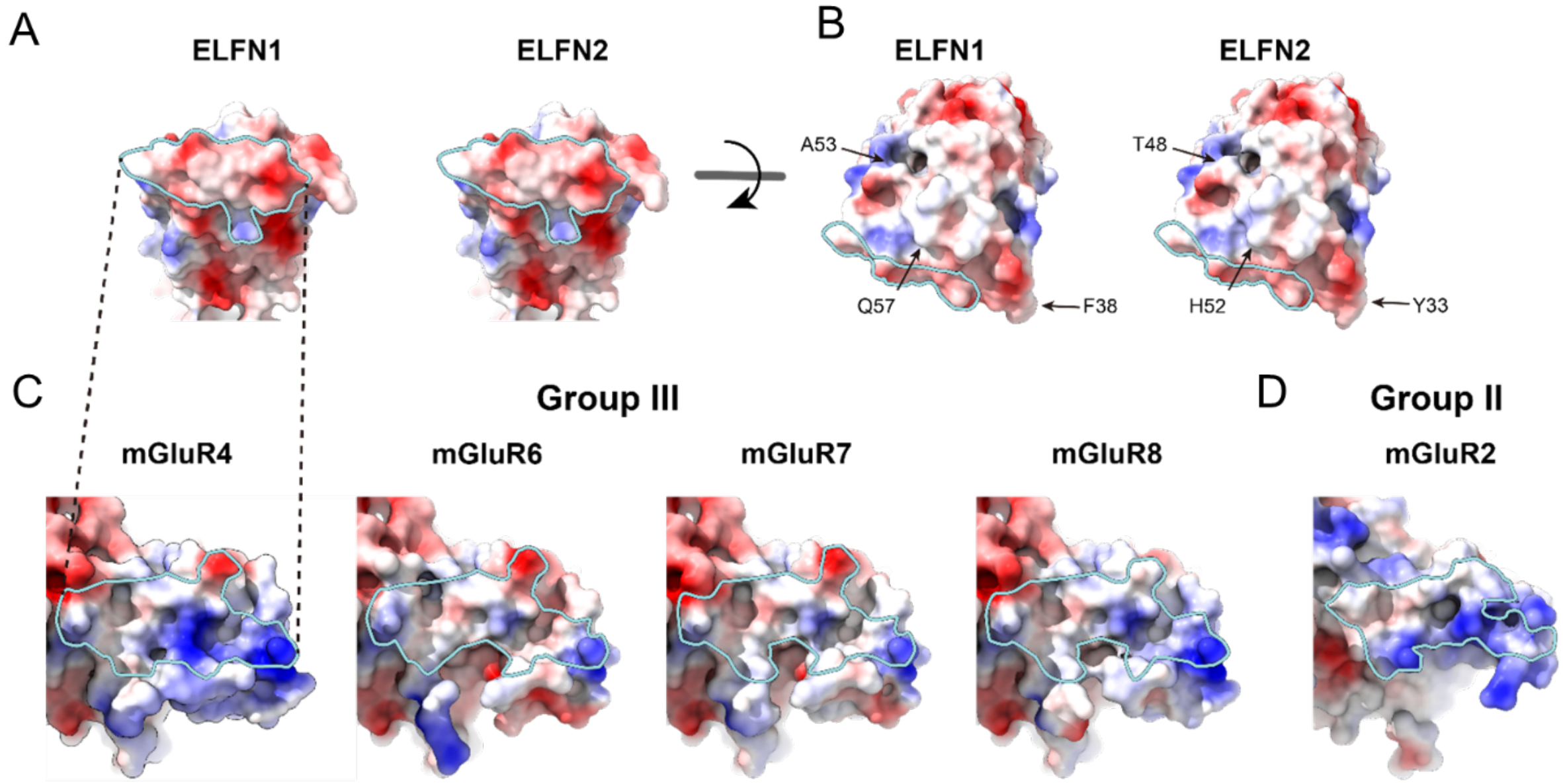

Supplementary Figure 7. ELFN-binding region is conserved between ELFNs and group III mGluRs.

A) Comparison of electrostatic landscape of ELFN1 vs. ELFN2 at mGluR interface. B) Comparison of non-conserved residues before ELFN2 N85 and ELFN1 N90. C) Comparison of electrostatic landscapes of group III mGluRs at ELFN interface. D) Comparison of group II mGluR2 (PDB: 7EPA) electrostatic landscape in the same region as ELFN interface.

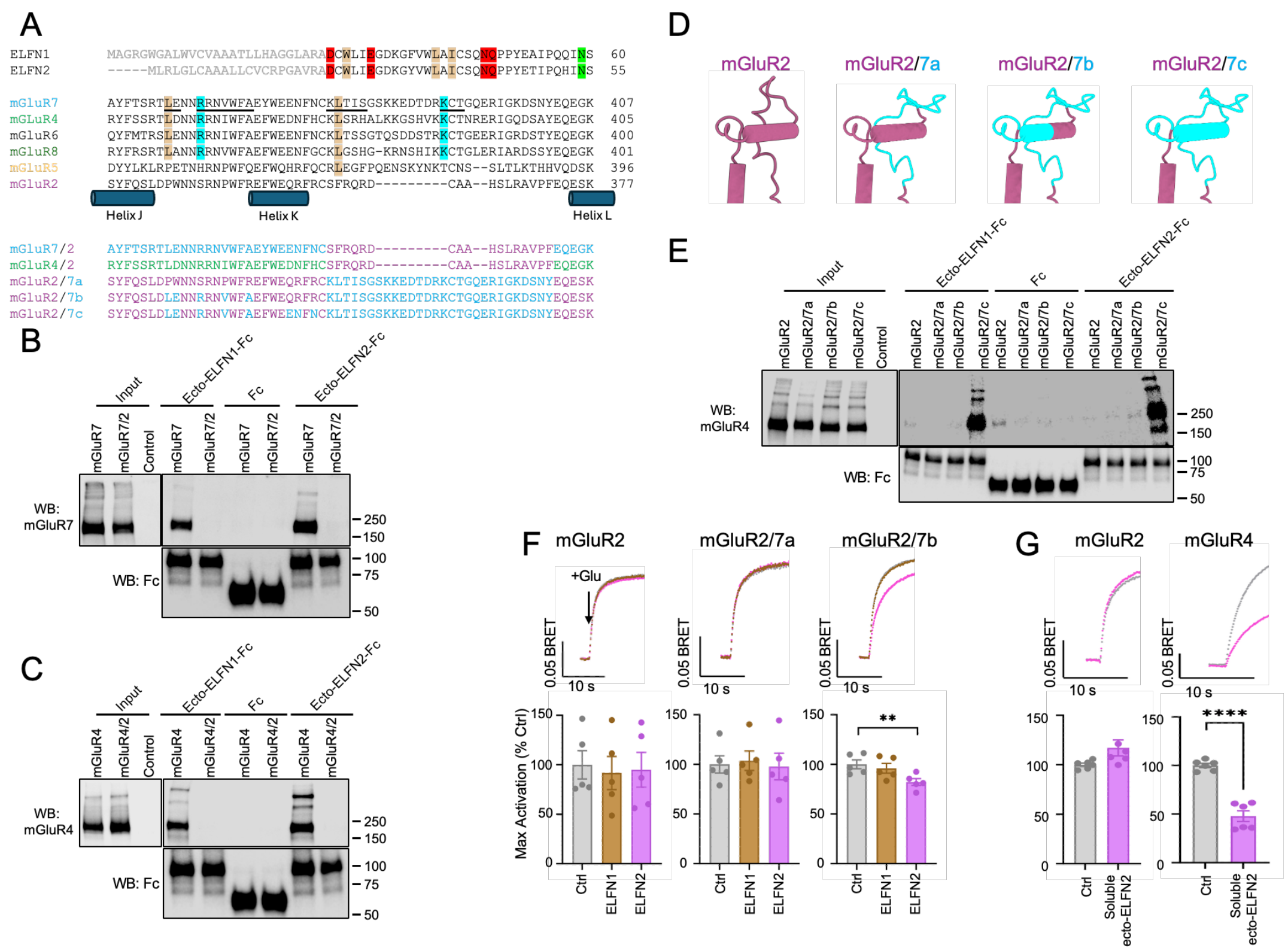

Supplementary Figure 8. Group III mGluR ELFN-binding region is sufficient to induce ELFN binding.

A) Sequence alignment of ELFNs and various mGluRs. Residues consevered in group III mGluRs that are involved in binding interface are highlighted brown – hydrophobic, red – acidic, blue – basic, green – glycosylated. Cleaved signal peptide is colored gray. Sequences of chimeric protein are also indicated. Residues equivalent to wild type mGluRs are colored blue – mGluR7, green – mGluR4, Brown – mGluR2. B) Representative immunoblots of wildtype and chimeric mGluR7 input (left), immunoprecipitation of ecto-ELFN1-Fc or ecto-ELFN2-Fc (bottom), and co-immunoprecipitation of mGluR7. Part of these data are also shown in Fig. 3B. C) Representative immunoblots of wildtype and chimeric mGluR4 input (left), immunoprecipitation of ecto-ELFN1-Fc or ecto-ELFN2-Fc (bottom), and co-immunoprecipitation of mGluR4. D) Cartoon depictions of wildtype and chimeric mGluR2. Residues derived from mGluR2 are colored red-brown and from mGluR7 are colored blue. E) Representative immunoblots of mGluR2 input (left), immunoprecipitation of ecto-ELFN1-Fc or ecto-ELFN2-Fc (bottom), and co-immunoprecipitation of mGluR2. F) Average change in BRET ratio of Gαo sensor activation by cells expressing wildtype or chimeric mGluR2 when co-cultured with ELFN- or control-expressing cells. Representative traces and max activation quantifications. Part of these data are also shown in Fig. 3D. Data are means ± SEM of five to six individual experiments (unpaired Student's t-test). G) Average change in BRET ratio of Gαo sensor activation by cells expressing indicated mGluR when treated with soluble ecto-ELFN2 or control. Representative traces and max activation quantifications. Data are means ± SEM of five to six individual experiments. *p<0.05; **p<0.01; ***p<0.001; ****p<0.0001 (unpaired Student's t-test).

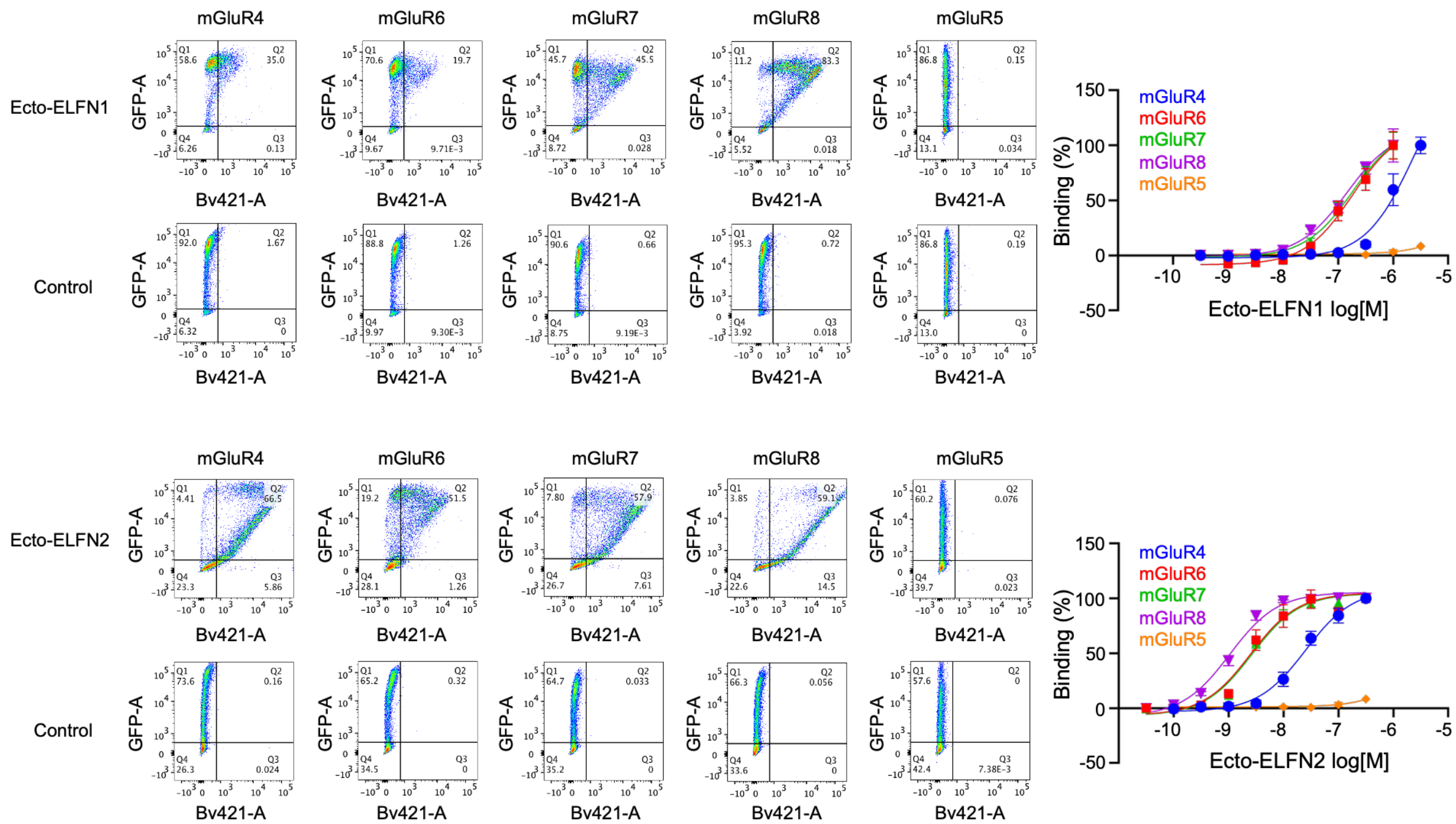

Supplementary Figure 9. Binding affinity hierarchy of ELFN and class III mGluR pairings. Representative Fluorescence-Activated Cell Sorting plots of ecto-ELFNs binding mGluRs. Soluble ecto-ELFN binding dose response curves for various mGluRs. Data are means ± SEM of five individual experiments.

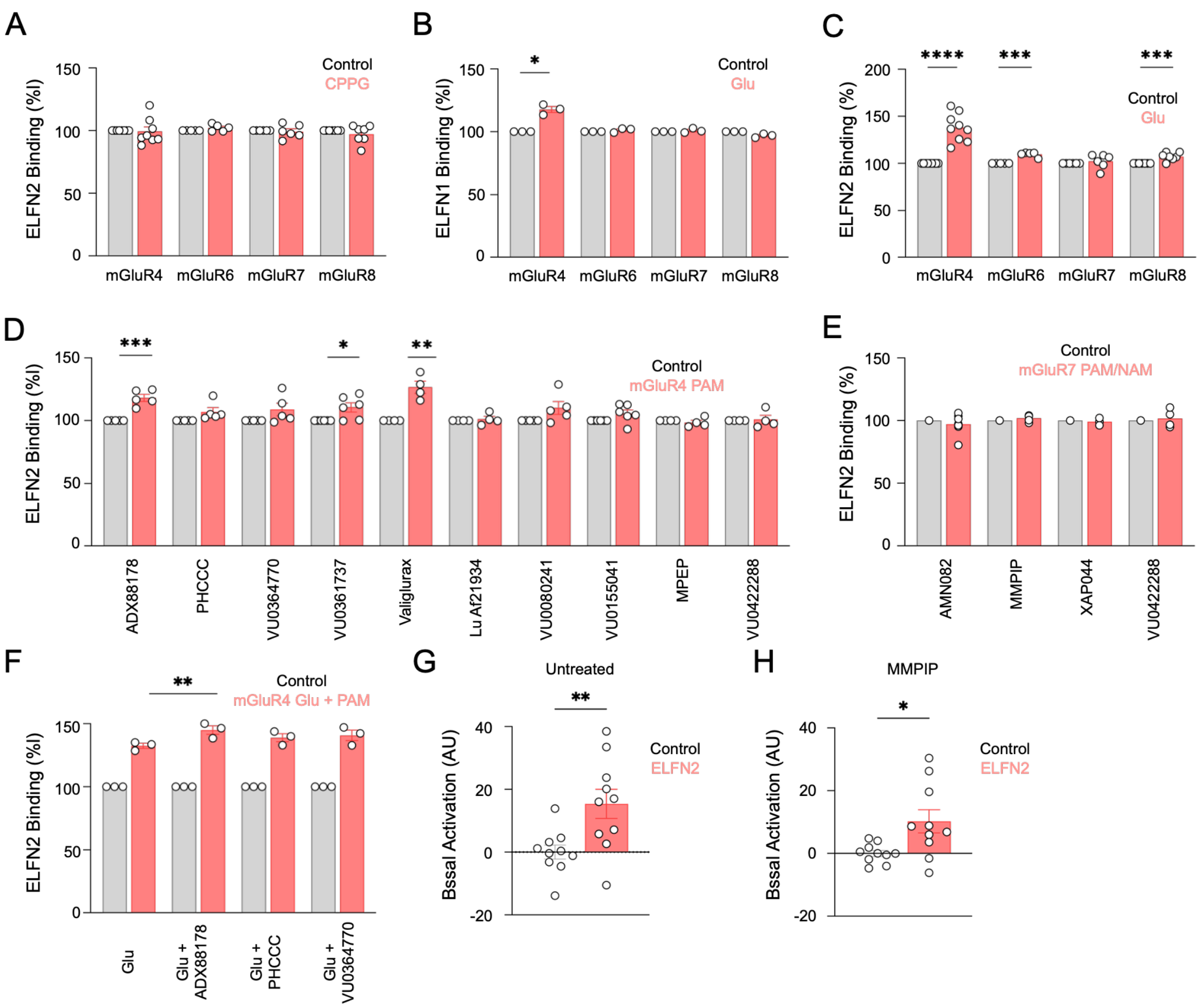

Supplementary Figure 10. Effect of additional mGluR ligands and allosteric modulators on ELFN binding affinity.

A) The effect of antagonist CPPG on max fluorescent intensity ELFN2 signal for each group III mGluR. Data are means ± SEM of eight individual experiments of three replicates each. B) The effect of glutamate on max fluorescent intensity ELFN1 signal for each group III mGluR. Data are means ± SEM of three individual experiments. C) The effect of glutamate on max fluorescent intensity ELFN2 signal for each group III mGluR. Data are means ± SEM of five to nine individual experiments of three replicates each. D) The effect of allosteric modulators on max fluorescent intensity ELFN2 signal for mGluR4. Data are means ± SEM of four to six individual experiments of three replicates each. E) The effect of allosteric modulators on max fluorescent intensity ELFN2 signal for mGluR7. Data are means ± SEM of four to seven individual experiments of three replicates each. F) The effect of allosteric modulators plus glutamate on max fluorescent intensity ELFN2 signal for mGluR4. Data are means ± SEM of three individual experiments of three replicates each. G) Average change in BRET ratio of Gαo sensor activation by unstimulated or H) MMPIP treated cells expressing mGluR7 when co-cultured with ELFN2- or control-expressing cells. Data are ± SEM of five individual experiments of two replicates each. *p<0.05; **p<0.01; ***p<0.001; ****p<0.0001 (unpaired Student's t-test).

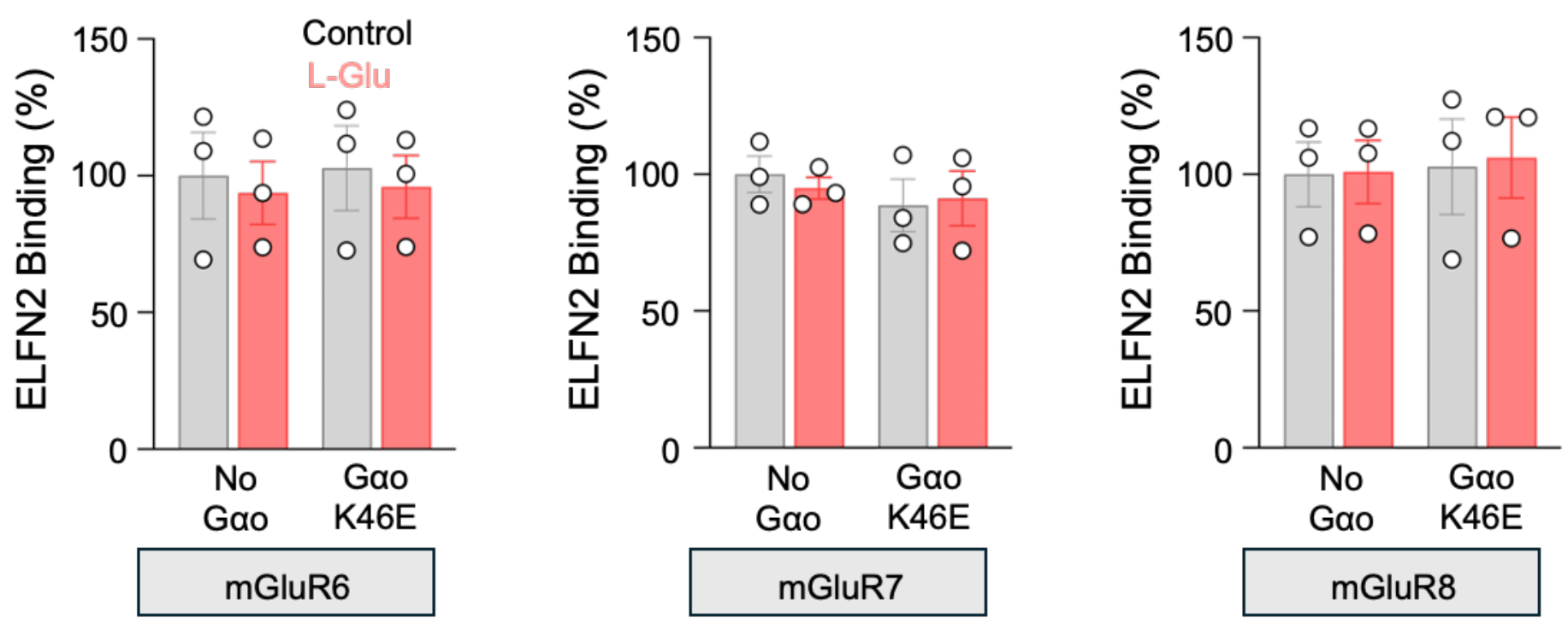

Supplementary Figure 11. Cooperativity of other group III mGluR with G proteins and ELFN.

Effect of Gαo K46E mutant binding on mGluR–ELFN affinity. Data are means ± SEM of three experiments. *p< 0.05; **p<0.01; ***p<0.001; ****p<0.0001. (Two-way Anova with Tukey's post hoc test)

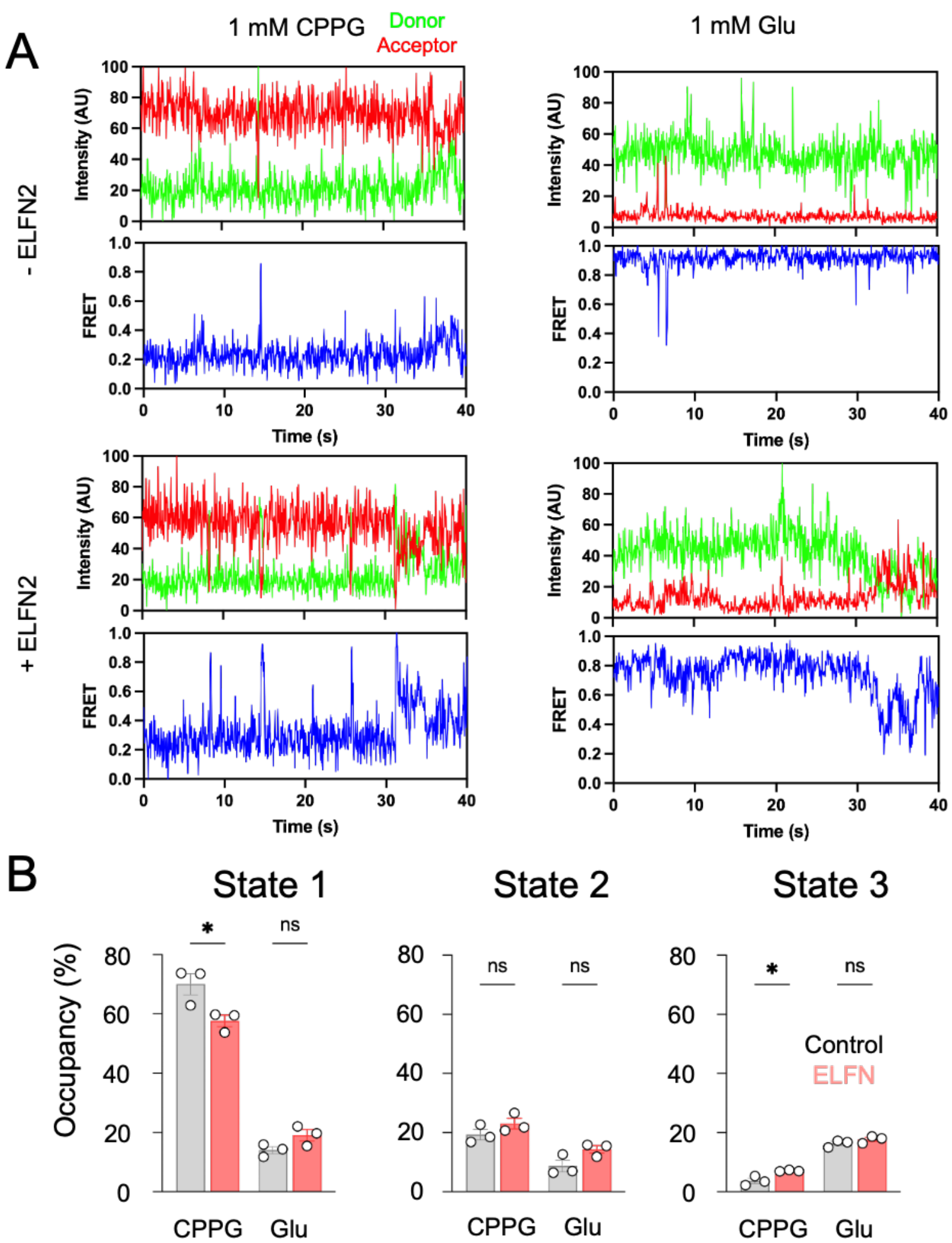

Supplementary Figure 12. ELFN2 alters occupancy of smFRET states

A) Representative smFRET traces of mGluR4 treated with glutamate, CPPG and/or ELFN2. Donor, acceptor, and FRET signals are shown in green, red, and blue. B) Occupancy of states 1–3. Data are means ± SEM of three experiments. *p< 0.05; **p<0.01; ***p<0.001; ****p<0.0001. (unpaired Student's t-test)

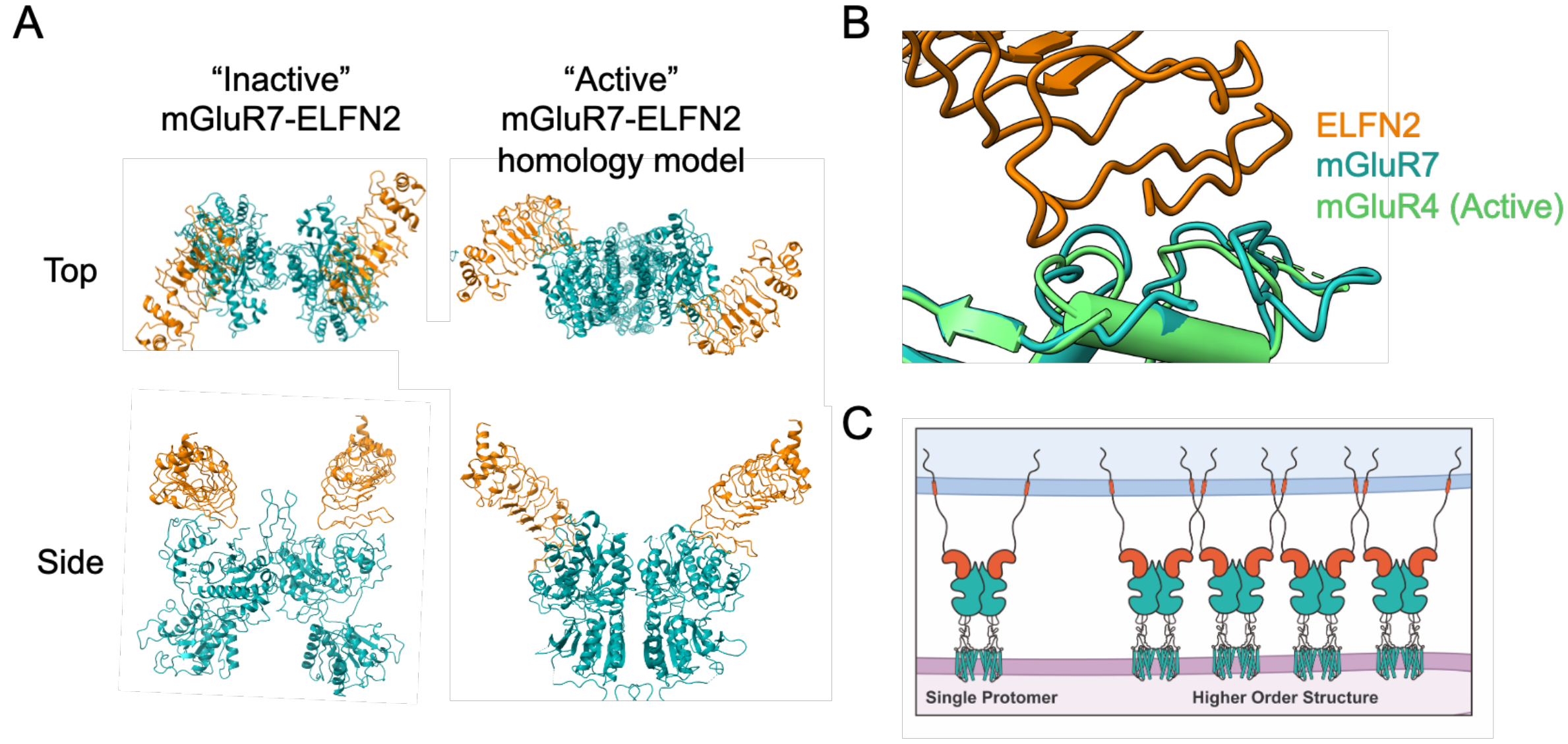

Supplementary Figure 13. Structural Prediction of Active mGluR-ELFN complex

A) Structural alignment of mGluR7-ELFN2 binding site onto structure of active mGluR4 (PDB:7E9H). B) Active mGluR4 shows no steric hindrance or deformation at ELFN binding site compared to mGluR7. C) Proposed model of higher order complex formation of alternating ELFN and mGluR dimers.

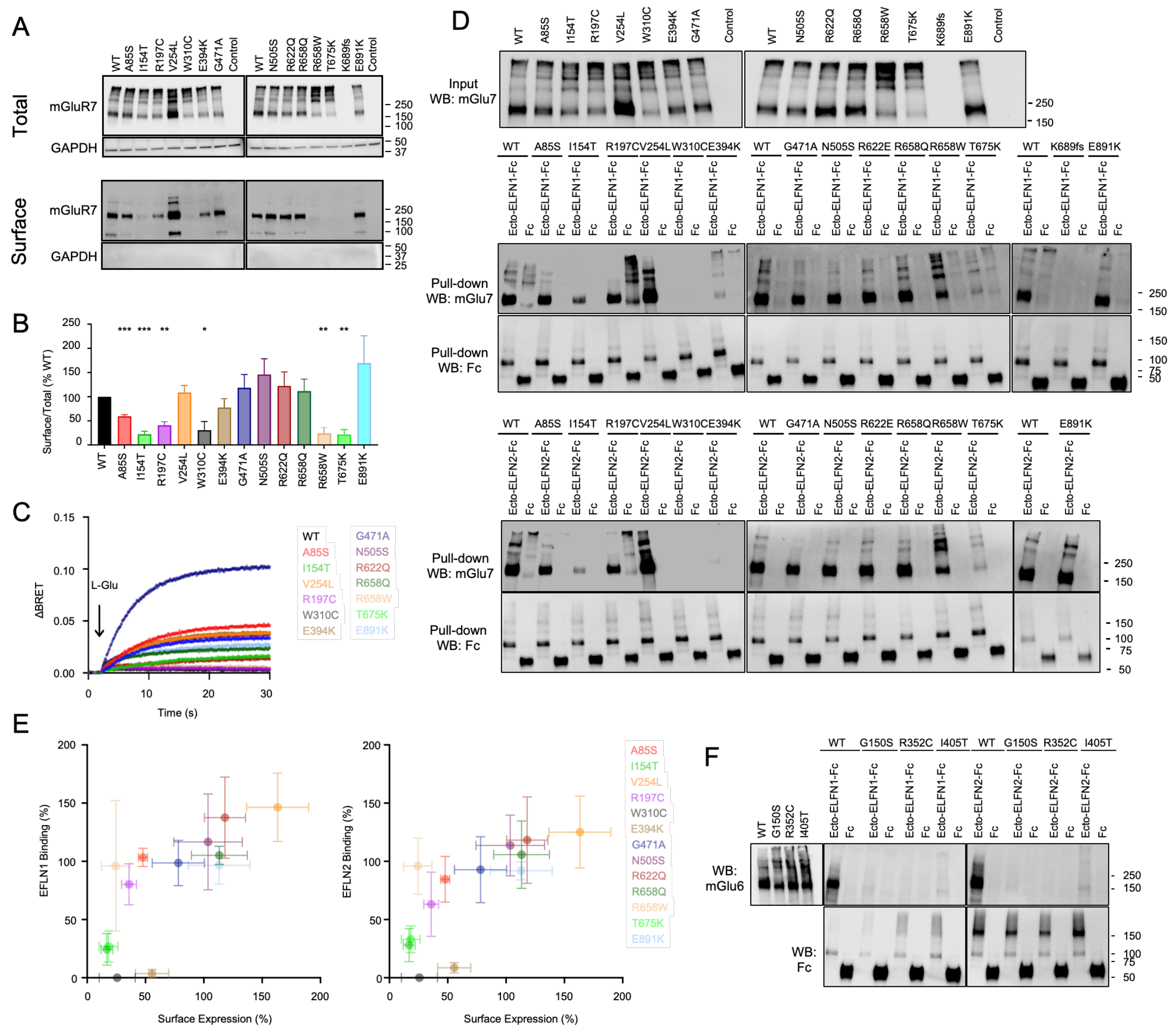

Supplementary Figure 14. Interference at the mGluR-ELFN Binding Interface is a Recurring Disease Mechanism.

A) Representative immunoblots of wildtype and mutant cell lysates and surface expressed mGluR7. Quantifications of total expression, surface expression, and the ratio of surface/total expression. B) Representative traces of change in BRET Gαo sensor activation in response to glutamate for each mGluR7 mutant. Data are means ± SEM of three individual experiments. C) Representative immunoblots of wildtype and mutant mGluR7 input D) Immunoprecipitation of ecto-ELFN1-Fc (row 3) or ecto-ELFN2-Fc (row 5) and co-immunoprecipitation of mGluR7 (rows 2 and 4). E) Plot of surface expression versus ELFN1 or 2 binding. Data are means ± SEM of three to four individual experiments. F) Representative immunoblots of wildtype and mutant mGluR6 input (left), immunoprecipitation of ecto-ELFN1-Fc (bottom left) or ecto-ELFN2-Fc (bottom right) and co-immunoprecipitation of mGluR7 (top). Part of these data are also shown in Fig. 7E. $^{*}p<0.05$; $^{**}p<0.01$; $^{***}p<0.001$. (unpaired Student's t-test)

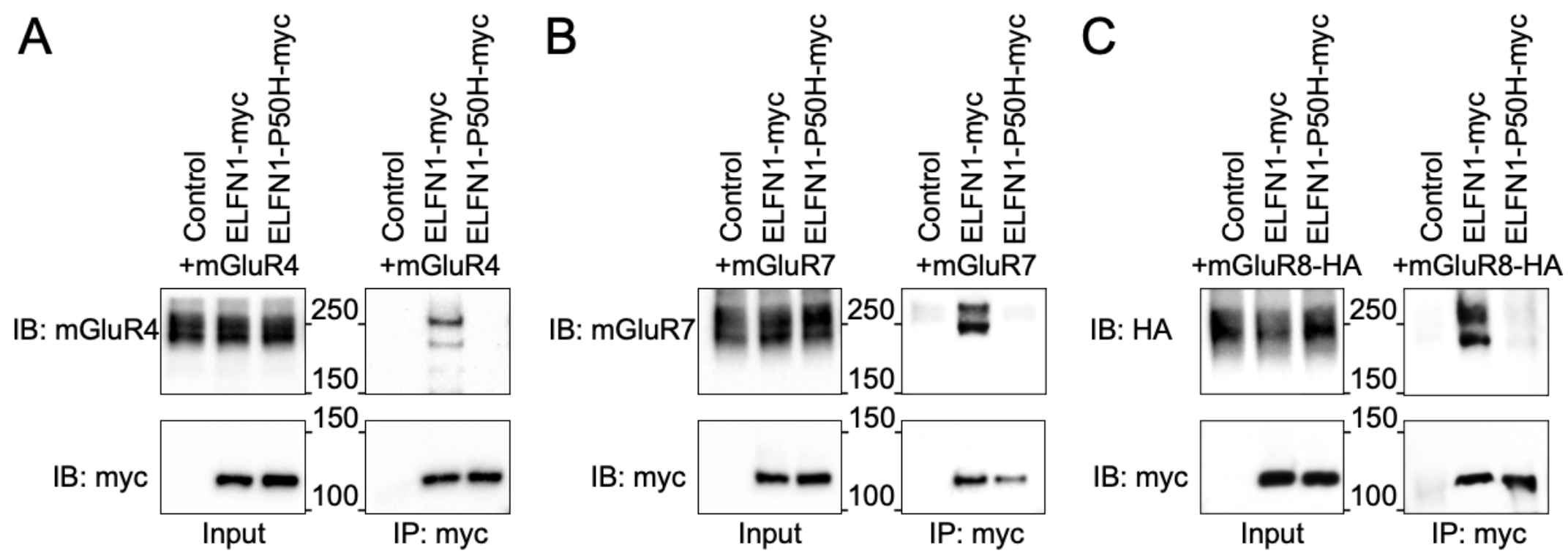

Supplementary Figure 15. ELFN1-P50H exhibits disrupted interaction with group III mGluRs in trans.

A) Representative immunoblots (IB) of mGluR4 and ELFN1-myc or ELFN1-P50H-myc inputs (left), and immunoprecipitation (IP) of ELFN1-myc or ELFN1-P50H-myc, and co-immunoprecipitation of mGluR4 (right). (B Representative immunoblots of mGluR7 and ELFN1-myc or ELFN1-P50H-myc inputs (left), and immunoprecipitation of ELFN1-myc or ELFN1-P50H-myc, and co-immunoprecipitation of mGluR7 (right). C) Representative immunoblots of mGluR8-HA and ELFN1-myc or ELFN1-P50H-myc inputs (left), and immunoprecipitation of ELFN1-myc or ELFN1-P50H-myc, and co-immunoprecipitation of mGluR8-HA (right).

Table S1. Cryo-EM data collection, refinement, and validation statistics.

	mGluR7 VFT–ELFN2 LRR (EMDB-72409) (PDB 9Y2F)
**Data collection and processing**	
Voltage (kV)	300
Electron exposure (e–/Å^2)	50
Defocus range (μm)	-1.0 to -2.0
Pixel size (Å)	0.75
Symmetry imposed	C2
Initial particle images (no.)	239,195
Final particle images (no.)	28,023
Map resolution (Å)	4.6
FSC threshold	
Map resolution range (Å)	4.6 to 6.4
**Refinement** (mGluR7 VFT–ELFN2 LRR)	
Initial model used (PDB code)	7EPC, AlphaFold
Model resolution (Å)	4.4
FSC threshold	
Model resolution range (Å)	4.3 to 4.6
Map sharpening *B* factor (Å^2)	-45
Model composition	
Non-hydrogen atoms	10,924
Protein residues	1,420
Ligands	0
B factors (Å^2)	
Protein	202.62
R.m.s. deviations	
Bond lengths (Å)	0.004
Bond angles (°)	0.605
Validation	
MolProbity score	2.26
Poor rotamers (%)	0.00
Ramachandran plot	
Favored (%)	89.09
Allowed (%)	10.91
Disallowed (%)	0.00

Movie S1. Conformation dynamics of mGluR7-ELFN2 complex.

Three-dimensional variability analysis (3DVA) of particles from the mGluR7-ELFN2 sample identifies populations of particles in the "Apo" inactive state with no ELFN2 bound, with only one ELFN2 monomer bound, with two ELFN2 monomers bound but still in the inactive conformation, and lastly with two ELFN2 monomers bound in the partially active state (as depicted in the main figures).
